# Virtual reality for experiential learning: enhancing agitation management skills, confidence, and empathy in healthcare students

**DOI:** 10.1080/10872981.2025.2542809

**Published:** 2025-08-06

**Authors:** Gabrielle Wann Nii Tay, Mian Mian Tong, John Yap, Hon Keat Mak, Shawn Yong Shian Goh, Cyrus Su Hui Ho

**Affiliations:** a Department of Psychological Medicine, Yong Loo Lin School of Medicine, National University of Singapore, Singapore, Singapore; b Application Architecture and Technology at NUS Information Technology, National University of Singapore, Singapore, Singapore; c Health and Social Sciences Cluster, Singapore Institute of Technology, Singapore, Singapore; d Department of Psychological Medicine, National University Hospital, Singapore, Singapore, Singapore

**Keywords:** Virtual reality, experiential learning, simulation-based learning, agitation management, empathy, confidence, patient care, healthcare education, psychiatry education

## Abstract

Agitation in healthcare, particularly in psychiatric settings, is a prevalent and escalating global concern. Despite its significance, healthcare students often feel underprepared to manage agitation, citing fear, stigma, and limited clinical exposure. Traditional teaching methods, such as lectures or simulations, are resource-intensive and offer limited opportunities for repeated practice in low-risk environments. Virtual reality (VR) offers a promising alternative, providing immersive, standardised, and repeatable training for high-stress clinical scenarios. In response, the education team at [redacted for peer review], developed the Managing AGgression using Immersive Content (MAGIC) programme. This three-hour blended learning workshop, a mandatory component of the psychiatry curriculum for medical and nursing students, integrates didactic teaching, role-play, and the Virtual Reality in Agitation Management (VRAM) activity. Through experiential learning, MAGIC aims to enhance healthcare students’ confidence, empathy, mental health literacy, and competence in managing agitation in psychiatric healthcare settings. Using a pre- and post-test quasi-experimental design, we evaluated the programme’s effectiveness among 152 medical and nursing students. Results demonstrated significant improvements in mental health literacy, self-perceived proficiency, and confidence in managing agitated patients; there was also a marked reduction in stigma towards individuals with mental health conditions. In addition, participants responded positively to all aspects of the VRAM software, underscoring its usability and educational value. These findings highlight the potential of integrating immersive VR technology with traditional pedagogical methods to transform healthcare education by fostering deeper engagement, enhancing clinical competence, and ultimately improving patient outcomes.

## Introduction

Patient agitation, characterised by excessive or inappropriate physical or verbal activity, remains a pervasive issue in healthcare [1]. Global prevalence rates reach up to 70.9% in Australia and New Zealand, 67.3% in North America, and 64.9% in Asia [2,3]. The COVID-19 pandemic has further exacerbated these concerns, contributing to a marked increase in agitation and violence against healthcare workers (HCWs) [4–6]. Such incidents significantly impact the mental and physical well-being of healthcare workers (HCWs) and compromise the quality of patient care [7].

While the behavioural management of agitation has received considerable attention, the development of HCWs’ empathy and communication skills – particularly among students – remains underexplored. This is especially relevant in psychiatry, where agitation often stems from conditions such as psychosis or mania and tends to present differently than in other medical specialities [8,9]. Agitated patients could challenge clinicians’ authority, highlighting the need for HCWs to possess empathetic and compassionate agitation management skills to ensure optimal patient outcomes [9].

Historically, agitation has been addressed using physical or chemical restraints [10–12]. Though often effective for immediate control, these methods also carry significant physical and psychological risks. For example, patients and HCWs alike may suffer physical injuries [13,14]; psychological risks include increased distress [15], cognitive decline [14], and disruption of therapeutic relationships [13], and such traumatic restraint experiences can also deter patients from seeking and complying with future care [16].

For HCWs, these encounters can lead to emotional trauma, burnout, and heightened job stress when inadequately equipped to manage agitated patients, all of which could contribute to high attrition. In Singapore, it was reported that the average turnover rate from 2020 to 2021 was approximately 7–9% for nurses and 3–5% for doctors in acute public hospitals [17]. While patient agitation can be a contributing stressor, broader systematic factors such as workplace conditions and organisational issues also play a substantial role in attrition [18,19]. A feedback loop may emerge where attrition worsens workloads and burnout, perpetuating further turnover and diminished care quality [20,21]. This highlights the need for approaches that prioritise empathy and de-escalation over coercion to promote safer healthcare environments and better HCW retention [22–24].

Empathy, a fundamental cognitive ability that enables HCWs to understand patients’ perspectives and respond with compassion, is central to therapeutic relationships and can significantly reduce reliance on physical or chemical interventions [25–28]. However, empathy is not consistently or explicitly taught in undergraduate healthcare curricula, particularly in psychiatry. Recent studies in Singapore show that while both medical and nursing students value psychiatry, many hold negative attitudes toward patients with mental illness and lack confidence in managing agitation, citing fear, stigma, and limited clinical exposure as key barriers [17,29–32].

Traditional training in empathy and communication training, such as lectures, case discussions, and simulated patients, is often infrequent, resource-intensive, and difficult to standardise [33,34]. These methods tend to depend heavily on instructor availability and vary in fidelity across teaching sites; they are also limited in their ability to meaningfully engage learners or recreate clinical encounters realistically [35]. Studies have also shown that passive or didactic formats may result in lower emotional involvement and reduced motivation to apply empathy-related skills in practice [36]. Moreover, these methods rarely allow repeated exposure to high-risk scenarios like patient agitation, nor do they provide psychologically safe environments to practise de-escalation skills [37,38]. As a result, learners may struggle to translate theoretical knowledge into confident, empathetic clinical practice.

Virtual reality (VR) technology has emerged as a promising alternative to these traditional approaches. By offering immersive, standardised, and repeatable simulations of realistic clinical scenarios, VR enables learners to develop communication, empathy, and decision-making skills in a low-risk environment. Compared to conventional methods, VR facilitates real-time engagement and storytelling-driven learning, which has been shown to enhance emotional connection and self-efficacy [39,40].

Empirical evidence supports the use of VR to improve clinical and interpersonal competencies [41]. VR technology provides a safe environment for learners to practise complex clinical scenarios [42], receive feedback, and build confidence in handling unpredictable, high-stress situations [43,44]. Notably, a randomised controlled trial conducted among mental healthcare professionals in Singapore found that participants who underwent a VR intervention reported significant reductions in stigma and improved attitudes toward individuals with psychotic disorders [45]. Another study focusing on the use of VR in empathy education for medical students also demonstrated that role-playing through VR improved some dimensions of empathy toward individuals with depression [46]. Taken together, these findings underscore the unique potential of VR to facilitate emotionally engaging, low risk learning experiences, thus making it particularly well-suited for preparing healthcare students to manage patient agitation with empathy and effectiveness. While VR is not without its limitations, such as higher technical costs and the need for pedagogical alignment, its strengths in standardisation, realistic replications of clinical scenarios, and safe skills practice address key gaps in conventional training [47].

From a practical standpoint, VR-based learning is more cost-effective and scalable than traditional pedagogical methods; it also supports self-directed learning, easing the burden on teaching faculty [48,49]. Its accessibility across time and location makes it especially useful for interdisciplinary and inter-institutional learning, ensuring equitable access to high-quality education [48,50]. Prior research also found that VR-based simulations not only improve learners’ confidence in clinical skills, but also enhance knowledge retention, satisfaction and emotional engagement [51–53].

To address the current healthcare education gap in agitation management, specifically in psychiatric settings, the education team at [redacted for peer review] developed the Virtual Reality in Agitation Management (VRAM) programme. VRAM provides learners with a safe, immersive, and standardised environment to repeatedly practise managing agitation. The scenarios are carefully designed to replicate time-sensitive, high-stakes clinical encounters, which include ethical dilemmas such as covert medication administration, mental capacity assessments, and prioritisation of care.

All fourth-year medical and second-year nursing students participated in the Managing AGgression using Immersive Content (MAGIC) programme, which combines the Empathetic Care and Response (ECARE) programme and VRAM. MAGIC comprises a video-aided didactic teaching session on managing agitation, tutor-led discussions, role-play sessions, and a tutor-facilitated debrief [54].

This study first investigates whether MAGIC enhances healthcare students’ self-perceived proficiency and confidence in managing agitation. It further examines the programme’s impact on improving their empathetic attitudes and mental health literacy. We hypothesise that students who complete MAGIC will report greater confidence in using de-escalation techniques and, when necessary, chemical and physical restraints. Additionally, we also expect them to demonstrate more empathetic and less stigmatising attitudes toward patients with mental health conditions compared to those who do not participate in the programme. To complement the evaluation of educational outcomes, this study also assesses the usability and acceptability of the VRAM software using the Virtual Reality Neuroscience Questionnaire (VRNQ). The VRNQ measures user experience factors such as immersion, comfort, and interface quality, which are critical for ensuring the VR technology’s feasibility and wider adoption in healthcare education.

## Methods

### Design

A pre- and post-test quasi-experimental design was implemented to examine the effects of using MAGIC on self-perceived proficiency and confidence in managing agitation among healthcare students. The study also sought to explore the impact of MAGIC on their empathetic responses and mental health literacy, particularly about managing patients with mental health conditions. Lastly, it investigated the confidence of healthcare students in initiating various techniques to manage agitation.

### The Managing Aggression Using Immersive Content (MAGIC) programme

The Managing Aggression using Immersive Content (MAGIC) programme was a mandatory component of the curriculum for fourth-year medical and second-year nursing students during their psychiatry rotations. Although students were enrolled in separate academic tracks, the curriculum was standardised across both groups. All programme materials were identical, and faculty across both tracks coordinated regularly to ensure consistent delivery and learning outcomes.

The MAGIC programme was delivered as a three-hour blended learning workshop comprising four components. Students first participated in a didactic lecture under the ECARE programme, introducing key concepts in agitation management. This was followed by a tutor-led session involving role-play exercises to practise communication strategies and physical restraint techniques. In the third segment, students engaged in a virtual reality simulation (VRAM), where they made real-time decisions in managing agitation within standardised, immersive clinical scenarios. The session concluded with a structured debrief facilitated by tutors using the RC22 model, which promotes reflective learning in healthcare simulation settings [55]. It encompasses several key stages – reaction, recollection, reflection, analysis, and application – essential for fostering a deeper understanding and integrating knowledge acquired from simulation-based learning [56].

The VRAM scenario was modelled on a paediatric ward hostage incident and refined using the VRAM team’s clinical experience, with all elements grounded in real encounters. In this scenario, the students assume the role of an on-call HCW managing a highly agitated female patient with drug-induced psychosis. The patient takes a young ward visitor hostage while demanding discharge against medical advice. While de-escalating the situation, the HCW [student] must manage concurrent tasks, including addressing an irate family member and responding to nursing staff demands. This immersive scenario simulates real-life pressures, requiring participants to prioritise and respond effectively under stress.

**Figure 1.**
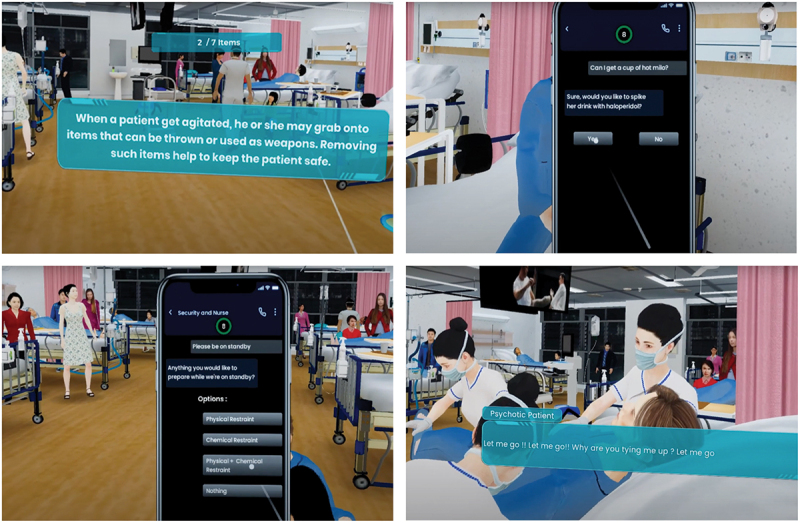
Examples of prompts and questions encountered by participants of VRAM.

This scenario was designed to train participants to recognise early signs of agitation, such as erratic behaviour and aggressive speech, and apply verbal de-escalation techniques effectively. Critical decisions must be made by the HCW [student] within an 8-second window; if no decision is made, the system selects a random action, mimicking the consequences of hesitation or delayed intervention in real-life clinical settings. Key challenges include covert medication administration, assessing mental capacity for discharge against medical advice, ensuring environmental safety by removing sharp objects, and coordinating nursing and security staff for physical and chemical restraint (see Figure 1).

Through this VRAM scenario, the students gain practical experience in crowd control and collaboration with nursing and security staff to implement physical restraint when necessary. Another crucial learning outcome is selecting and administering appropriate rapid tranquillisation medications. This immersive, real-time simulation enables students to practise essential agitation management skills in a safe and controlled environment, equipping them for similar clinical challenges. Decisions made during the scenario can lead to varied outcomes: poor choices may escalate agitation and increase the risk of harm. In contrast, well-executed decisions – such as timely medication administration and effective teamwork – can soothe the agitated patient and restore ward safety.

### Data collection

Participation in this quasi-experimental research study was voluntary for medical and nursing students enrolled in the compulsory MAGIC programme during their psychiatry rotation. Recruitment occurred immediately before the programme began and was thus conducted separately for medical and nursing students. All students were assured that non-participation would not affect their academic standing or programme completion. Ethical approval was obtained from the [redacted for peer review], and informed consent was obtained from all participants prior to data collection. Data were collected from August 2021 to July 2022 using pre- and post-programme questionnaires, with all responses anonymised. Although the cohorts were relatively homogenous in age and year of study – medical students were primarily fourth-year students aged around 22 to 25, while nursing students were mainly second-year students aged 20 to 25 — gender and ethnicity data were collected for transparency and reported in the Results. Post-programme questionnaires were administered immediately following completion of the MAGIC programme.

Measures included the Jefferson Scale for Empathy (JSE), Opening Mind Scales for Healthcare Providers (OMS-HC-15), and the Mental Health Literacy Scale (MHLS). Participants also assessed their self-perceived competency and confidence in managing situations involving agitated patients and in communicating with such individuals.

The JSE is a widely recognised 20-item questionnaire developed by Hojat and colleagues to measure healthcare professionals’ attitudes towards empathy [57]. Each item on the JSE is rated on a 7-point Likert scale, with higher scores indicating more empathetic attitudes. The JSE has demonstrated strong internal consistency, with Cronbach’s alpha coefficients averaging 0.80 [58,59]. Numerous studies have also validated the JSE in various cultural contexts, with translations available in multiple languages, including Chinese and Turkish [60,61]. It has been shown to effectively capture the nuances of empathy in healthcare settings.

The MHLS evaluates knowledge of various aspects of mental health, along with attitudes towards mental health and help-seeking. It comprises 35 items, each rated on a 4 or 5-point Likert scale, with a higher total score indicating a greater level of mental health literacy. It has shown strong internal consistency, with Cronbach’s alpha coefficients reported to be approximately 0.85 [62]. The questionnaire also correlates well with other established measures of mental health literacy [63], thereby supporting its validity.

In contrast, a higher score on the 15-item OMS-HC-15 questionnaire, which employs a 5-point Likert scale, indicates more stigmatising attitudes toward individuals with mental health conditions. The OMS-HC-15 was developed to evaluate healthcare providers’ attitudes and behavioural intentions, including their willingness to disclose and seek help [64,65]. It has demonstrated strong internal consistency, with Cronbach’s alpha coefficients around 0.82 [66]. It has also demonstrated a strong correlation with other established measures of stigma and attitudes towards mental health [65]; furthermore, a clear factor structure aligned with the theoretical framework of stigma was revealed during the validation of the questionnaire [67]. Therefore, lowered OMS-HC-15 scores after an intervention reflect a positive shift towards less stigma.

The Virtual Reality for Neuroscience Questionnaire (VRNQ) was included in the post-MAGIC assessment to evaluate participants’ perceptions of VRAM, including user experience, game mechanics, in-game assistance, and virtual reality-induced symptoms and effects (VRISE). Each sub-domain comprised five items scored individually using a 7-point Likert scale. Higher scores indicated better quality (e.g., greater ease of use, how helpful an element of the software was, etc.) in all domains except VRISE, where higher scores indicated greater symptom intensity (e.g., nausea, disorientation, and instability). The VRNQ demonstrates strong internal consistency (Cronbach’s alpha coefficients typically exceed the acceptable threshold of 0.70) [68]. The VRNQ has been validated, as it correlates well with other established measures of user experience and cybersickness and can comprehensively assess the quality of VR software and the intensity of VR-induced symptoms [69].

### Data analysis

All data were analysed using IBM SPSS version 28.0. All statistical tests were two-tailed, with a significance level of *p* < 0.05. Both paired samples *t*-tests and independent t-tests were employed to compare continuous variables (e.g., mean questionnaire scores before and after MAGIC, mean VRNQ scores of medical and nursing subgroups, etc.).

Two cut-off scores were calculated to assess the suitability of VRAM – the minimum cut-off score was 25 for each sub-domain and 100 for the overall VRNQ score (i.e., a median rating of at least 5 on each item, corresponding to ‘High’), while the parsimonious cut-off score was 30 for each sub-domain and 120 for the overall VRNQ score (i.e., a median rating of at least 6 on each item, corresponding to ‘Very High’) (Kourtesis et al., 2019). These cut-offs ensure the safety and appropriateness of the VRAM software for use in medical education.

## Results

### Participant characteristics

The responses to the pre-and post-MAGIC questionnaires from 152 participants who provided complete data were analysed. Of these, more than half (60.6%) were female, and the majority identified as ethnically Chinese (90%). 69.1% (*N* = 105) were medical students, with the remainder being nursing students.

### Opening minds scale for healthcare providers, mental health literacy scale, and Jefferson scale for empathy

**Figure 2.**
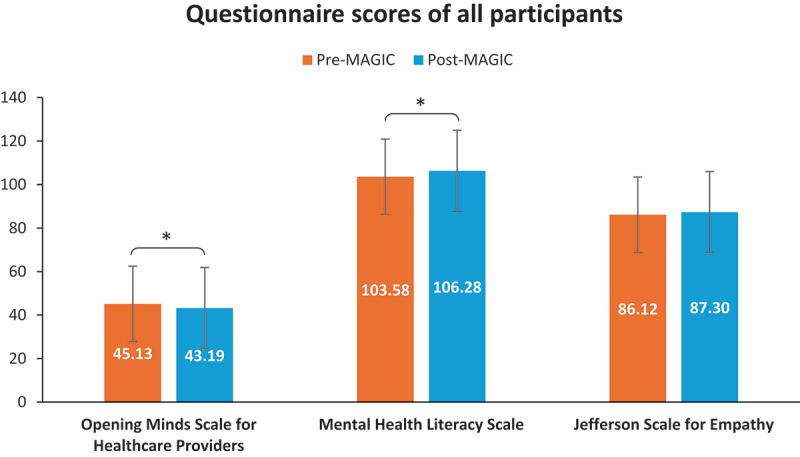
Comparison between participants’ (a) Opening minds scale for healthcare providers (OMS-HC-15), (b) mental health literacy scale (MHLS), and (c) Jefferson scale for empathy (JSE) questionnaire scores pre- and post-MAGIC.

There was no significant difference in participants’ scores for the Jefferson Scale for Empathy (JSE) questionnaire after they completed the MAGIC programme. However, statistically significant differences were observed in the scores for both the Opening Minds Scale for Healthcare Providers (OMS-HC-15) and Mental Health Literacy Scale (MHLS) questionnaires before and after they completed the MAGIC programme (see Figure 2).

Post-MAGIC MHLS scores (mean = 106.28, SD = 60.68) were significantly higher than pre-MAGIC MHLS scores (mean = 103.58, SD = 48.10, *t* = −3.59, *p* < 0.001), indicating an improvement in mental health literacy. Conversely, post-MAGIC OMS-HC-15 scores (mean = 43.19, SD = 20.35) were significantly lower than those reported pre-MAGIC (mean = 45.13, SD = 16.94, *t* = −1.64, *p* < 0.001), reflecting a significant reduction in stigmatising attitudes. Post-MAGIC JSE scores (mean = 87.30, SD = 45.96) were not significantly different from pre-MAGIC JSE scores (mean = 86.12, SD = 41.12, *t* = 3.86, *p* > 0.05).

Separate analyses of scores from the medical and nursing student subgroups yielded results like those of the overall sample (see Supplementary Figure S1). Further analyses also showed that the post-MAGIC scores for all three questionnaires did not differ significantly between the medical and nursing students.

### Confidence in managing agitation

**Figure 3.**
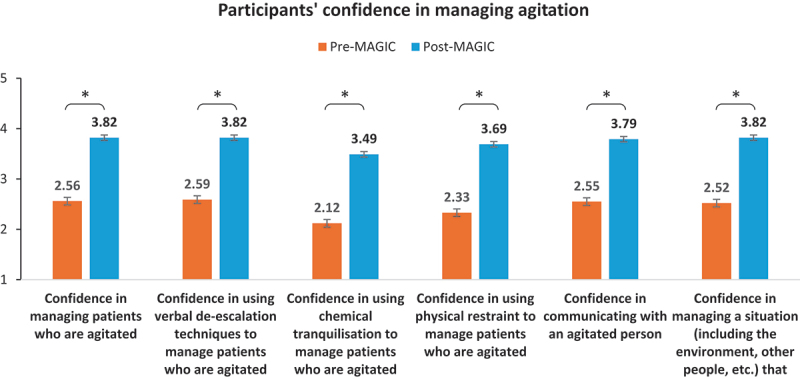
Comparison between confidence levels of participants (as measured based on a 7-point Likert scale) pre- and post-MAGIC.

Participants reported feeling more competent and confident in communicating with and managing agitated patients or situations involving them after undergoing the MAGIC programme, as evidenced by the significantly higher confidence levels reported post-MAGIC (see Figure 3).

Participants generally reported feeling more confident in managing patients who are agitated after experiencing MAGIC (mean = 3.82, SD = 1.24), with significantly higher confidence levels compared to their pre-MAGIC ratings (mean = 2.56, SD = 1.24, *t* = −11.12, *p* < 0.001). Similarly, they also reported increased confidence in communicating with an agitated person post-MAGIC (mean = 3.79, SD = 1.03), showing significantly higher confidence levels than their pre-MAGIC ratings (mean = 2.55, SD = 1.23, *t* = −11.37, *p* < 0.001).

Medical students, however, tended to be more confident in managing agitated patients and situations involving an agitated individual than nursing students (see Supplementary Figure S2). This is evident in the significant differences between student subgroups in pre- and post-MAGIC confidence ratings.

### Experiences with virtual reality in agitation management (VRAM)

**Figure 4.**
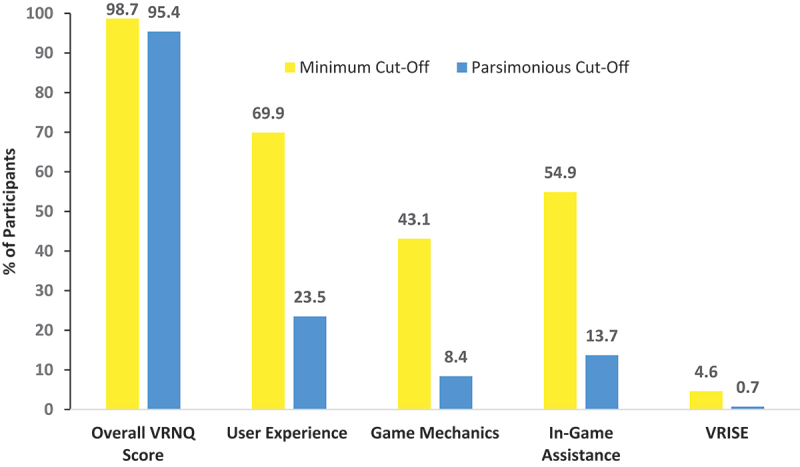
Percentage of participants whose scores met the minimum and parsimonious cut-offs for both the virtual reality Neuroscience questionnaire (VRNQ) sub-domains and overall scores. *VRISE = virtual reality induced symptoms and effects.

Participants also responded positively to various aspects of the VRAM component of MAGIC, with 98.6% and 95.4% of VRNQ total scores meeting the minimum (≥100) and parsimonious (≥120) cut-off scores, respectively (see Figure 4).

The percentage of participants whose scores for the User Experience, Game Mechanics and In-Game Assistance sub-domains meet the minimum ( >20) and parsimonious ( >30) cut-offs, indicative of a median rating of 5 (‘High’) per questionnaire item across these aspects of the VRAM software, which reflects the software’s adequacy regarding its suitability and ease of use. Furthermore, only 4.6% and 0.7% of VRISE sub-domain scores met the respective minimum and parsimonious cut-offs, indicating that the VRAM software is suitable for use without significant VRISE accompanying it.

**Figure 5.**
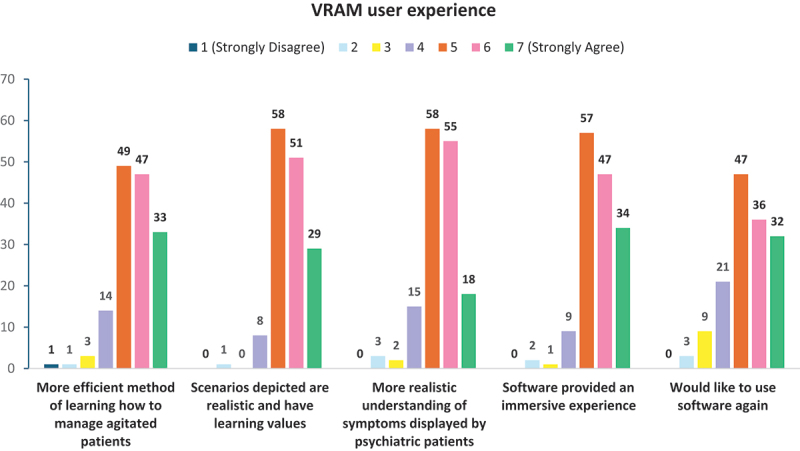
Distribution of participants’ ratings (on a 7-point Likert scale) of statements elucidating their experiences with using the VRAM software.

Participants generally had positive experiences with the VRAM software, with 90.8% (*N* = 138) agreeing that the scenarios portrayed are realistic and possess educational value (see Figure 5). Furthermore, 84.9% (*N* = 129) concurred that the software provided a more effective method for learning agitation management skills than traditional approaches, such as didactic lectures. Additionally, 75.7% (*N* = 115) expressed a desire to reuse the software.

Overall, there were no significant differences in the distribution of responses regarding their VRAM user experience between the subgroups of medical and nursing students.

### Evaluation of the MAGIC program

**Figure 6.**
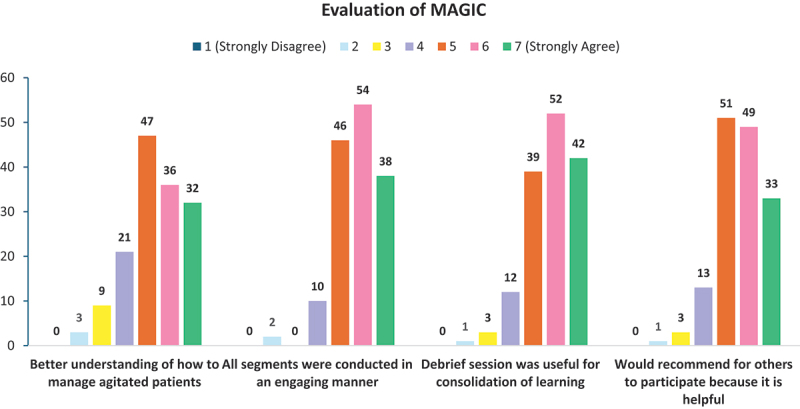
Distribution of participants’ ratings (on a 7-point Likert scale) of statements evaluating the MAGIC program.

Approximately 75% of participants (*N* = 115) agreed that the MAGIC programme gave them a better understanding of managing agitated patients. In comparison, 87.5% (*N* = 133) stated that they would recommend others to participate because they found it helpful (see Figure 6).

Additionally, 90.8% (*N* = 138) of participants agreed that all programme segments were engaging. In comparison, 87.5% (*N* = 133) felt that the tutor’s debrief segment helped consolidate their learning. An analysis of the distribution of responses regarding their evaluation of the MAGIC programme revealed no significant differences between the medical and nursing student subgroups.

## Discussion

Participants responded positively to both the VRAM component and the overall MAGIC programme. Most agreed that VRAM provided a more effective approach to learning agitation management, citing the realistic scenarios and embedded learning points as key strengths. These findings support previous studies, which have shown that authentic learning environments enhance meaningful learning and better prepare students for clinical challenges [49,70]. Olufunke et al. [71] also posit that realism in education is vital in promoting a deeper understanding and knowledge retention by increasing learner motivation through relevant, practical experiences [71].

Participants also reported significant improvements in mental health literacy, notable reductions in stigma, and increased confidence and self-perceived competency in managing agitation in psychiatric settings. Many shared that the MAGIC programme enhanced their understanding of agitation management and expressed a strong willingness to recommend it to peers, recognising its value in strengthening knowledge and practical skills. These findings are discussed in more detail below.

Post-MAGIC improvements in MHLS scores indicate enhanced understanding of mental health conditions, while reductions in OMS-HC-15 scores reflect decreased stigmatising attitudes since higher OMS scores represent more stigma. Previous research suggests that practical experiences significantly enhance mental health literacy by helping healthcare students recognise symptoms, understand treatment options, and appreciate the complexities of mental health conditions [72,73]. For example, Seow et al. [72] emphasise that clinical exposure deepens students’ comprehension of mental health and the intricacies of patient interaction, ultimately improving patient care. This likely explains the participants’ improvements in MHLS scores, as they may better appreciate and understand the complexities of mental health conditions, including the social, cultural, and environmental factors that influence patient experiences, through their interactions with the various components of the VRAM scenario. Yang et al. [74] also found that personal experiences with mental health conditions correlate positively with mental health literacy [74]. Considering that the VRAM segment of MAGIC allowed participants to engage with a simulated agitated patient who has drug-induced psychosis, it may have helped them better appreciate patients’ struggles and understand their needs and experiences more [75,76].

Moreover, as participants progressed through MAGIC, their biases and misconceptions about mental health could have been challenged, leading to positive shifts in attitudes. Research shows that nursing students on mental health clinical placements frequently report increased confidence and more positive attitudes towards mental health nursing [77,78]. Other studies also discovered that simulation experiences can reduce stigma and discrimination against individuals with mental health conditions [78,79]. These attitudinal shifts are crucial for reducing stigma and improving the quality of care for these patients.

Given the brief three-hour interval between the pre- and post-MAGIC assessments, the lack of significant change in empathy scores on the JSE was anticipated. Empathy is a complex trait that develops through sustained exposure to empathy-building interventions [80,81]. As a single, three-hour programme, MAGIC alone may not suffice to produce measurable shifts in empathetic understanding or behaviour. Additionally, individual factors such as baseline empathy, personality, and prior experiences could significantly influence empathy scores. For instance, individuals with high baseline empathy tend to show less variability in scores, making it challenging to observe changes following short-term interventions [82,83]. Nonetheless, MAGIC provides a valuable foundation for fostering empathy by introducing essential concepts and strategies related to empathetic understanding and behaviour. This foundational knowledge could enhance participants’ comprehension of patient perspectives and catalyse the development of deeper empathetic skills over time as they progress through their clinical training.

Participants also reported significant increases in self-perceived competence and confidence in managing agitated patients and communicating effectively after participating in MAGIC. This improvement may be attributed to the VRAM’s capacity to offer immersive and experiential learning opportunities, which reinforced understanding beyond traditional didactic methods [84]. The Cognitive Affective Model of Immersive Learning (CAMIL) posits that immersive VR can facilitate the acquisition of factual, conceptual, and procedural knowledge, thereby enhancing the learning experience and promoting skill transfer [85]. Simulation-based training, including VR, has consistently demonstrated effectiveness in boosting medical and nursing students’ confidence and clinical competence [84,86]. VR simulations are especially valuable for developing critical decision-making skills in high-pressure situations by immersing learners in realistic scenarios that demand time-sensitive responses to patient agitation. VR training fosters the development of rapid assessment and intervention abilities, with various studies underscoring the effectiveness of experiential learning, such as simulations, in raising students’ competencies and confidence in clinical settings [47,87,88]. [86] further reinforce this, demonstrating that simulation-based training significantly enhances knowledge and performance in managing acutely agitated psychiatric patients.

Repeated exposure to simulated scenarios with agitated patients helps solidify understanding, sharpen decision-making, and improve overall competence in managing challenging situations [39,42]. Prior studies have also shown that VR training significantly improves students’ efficiency in mastering specific medical techniques, reinforcing confidence and skill through repeated practice [89,90]. Furthermore, Hudgins et al. [91] found that nursing students reported increased confidence after participating in psychiatric care-based simulations, highlighting the value of practical experience in building self-assurance. By offering opportunities for practice and skill refinement in a controlled virtual environment, integrating VR with didactic teaching could empower students to approach real-world clinical encounters with greater confidence in their competencies as HCWs.

Furthermore, VR simulations can help reduce the anxiety students often experience when facing fundamental patient interactions, particularly in managing agitation. By immersing learners in realistic simulations, VR supports the development of effective coping strategies and emotional regulation in stressful situations. This is particularly crucial in psychiatric settings, where unpredictable patient behaviour can be overwhelming for novice healthcare providers [91,92].

The overwhelmingly positive responses to the VRNQ and the minimal reports of VRISE strongly suggest that VRAM is an engaging, effective, and safe educational tool [68,69]. Participants praised its user experience, game mechanics, and in-game assistance, highlighting its well-designed and user-friendly nature. These findings demonstrate the potential of VRAM as a valuable educational resource for acquiring clinical skills and knowledge in a safe and efficient manner. Expanding VRAM to simulate diverse clinical scenarios could create a versatile learning platform for general psychiatric healthcare education, enabling healthcare professionals to develop broader clinical skills and knowledge, ultimately enhancing patient care.

VRAM represents a pioneering approach in psychiatric medical education, complementing traditional didactic teaching methods to offer a more holistic learning experience. The VRAM simulation can notably enhance medical and nursing students’ confidence in managing agitation, a critical skill for reducing healthcare resource utilisation and improving patient outcomes [7,93]. By practising in a safe, virtual environment, students can hone their skills without the fear of causing harm or the pressure to always make the ‘right’ decisions, ultimately fostering greater competence in fundamental clinical interactions. Additionally, the requirement to make real-time decisions during the VRAM scenario enables ongoing reflection on their performance as the simulation progresses. This feedback loop reinforces learning, helps identify areas for improvement, and cultivates the critical thinking essential for managing agitation effectively [94]. However, it would be essential to recognise that VR simulations may not fully capture the complexities of real-world clinical settings. This limitation can restrict students’ exposure to the full range of patient interactions and scenarios, potentially hindering the development of comprehensive clinical skills. Furthermore, the effectiveness of VR learning varies among individuals, influenced by individual preferences, learning styles, and comfort with the technology [95]. Some learners may find VR experiences disorienting or overwhelming, which could negatively impact their overall learning experience. While these findings highlight the educational value of VRAM in enhancing agitation management skills, they should be interpreted cautiously given the following limitations.

First, as all fourth-year medical and second-year nursing students were required to participate in MAGIC as part of their curriculum, a control group could not be established, thus limiting causal interpretation of the findings. Second, post-assessments were conducted immediately after the session, with no longitudinal follow-up to assess sustained impact. Third, all outcomes were self-reported, introducing potential bias. Fourth, using a single VR scenario may limit generalisability across clinical contexts. Fifth, data from medical and nursing students were combined, which may have masked differences between groups. While this approach aligned with the programme’s interdisciplinary design and reflected the sample distribution, medical students typically receive more training in theoretical and pharmacological aspects of care, whereas nursing curricula often emphasise practical skills such as behavioural management and therapeutic communication. These differing training emphases could have influenced how students engaged with and benefited from participating in MAGIC. Lastly, while basic sociodemographic data such as sex and ethnicity were collected, the relative homogeneity of the sample limited the feasibility and value of subgroup analyses. Future studies should consider including a control group, conducting longer-term follow-up, using objective outcome measures, introducing multiple VR scenarios, and performing subgroup analyses comparing medical and nursing students.

Overall, MAGIC seeks to lay a solid foundation for addressing the pervasive stigma around mental health and enhancing the competency of healthcare workers in managing patients with mental health conditions. The findings of this study underscore the importance of experiential learning in preparing healthcare professionals for real-world challenges in psychiatric healthcare, as it deepens their educational experiences with increased knowledge and practical skills. The notable improvements in mental health literacy, self-perceived proficiency, and confidence in handling agitated patients demonstrate the value of incorporating and blending VR into the existing psychiatric healthcare education framework by equipping medical and nursing students with these essential skills and fostering a culture of empathy that extends beyond the immediate training environment. Furthermore, we hope to reduce burnout and attrition by equipping healthcare workers with comprehensive skills in agitation management, contributing to a more sustainable and effective healthcare workforce.

## Supplementary Material

VRAM Manuscript_MEO_Supp Material_17 Jun.docx

## Abbreviations

MAGIC: Managing AGgression using Immersive Content
VR: Virtual reality
VRAM: Virtual Reality in Agitation Management
HCWs: Healthcare workers
ECARE: Empathetic Care and Response
JSE: Jefferson Scale for Empathy
OMS-HC-15: Opening Mind Scales for Healthcare Providers
MHLS: Mental Health Literacy Scale
VRNQ: Virtual Reality for Neuroscience Questionnaire
VRISE: Virtual reality-induced symptoms and effects
