# Correction

**DOI:** 10.1080/10872981.2025.2547507

**Published:** 2025-08-18

**Article title**: Virtual reality for experiential learning: enhancing agitation management skills, confidence, and empathy in healthcare students

**Authors**: Gabrielle Wann Nii Tay, Mian Mian Tong, John Yap, Hon Keat Mak, Shawn Yong Shian Goh and Cyrus Su Hui Ho

**Journal**: *Journal of* Medical Education Online

**DOI**: https://doi.org/10.1080/10872981.2025.2542809

The author requested minor revisions to the published article. The changes are listed below:

- **In abstract**: The sentence “*In response, the education team at [redacted for peer review], developed the Managing AGgression using Immersive Content (MAGIC) programme.”* has been updated to *“In response, the education team at the Yong Loo Lin School of Medicine, National University of Singapore, developed the Managing AGgression using Immersive Content (MAGIC) programme.”*
- **In introduction**: The sentence *“To address the current healthcare education gap in agitation management, specifically in psychiatric settings, the education team at [redacted for peer review] developed the Virtual Reality in Agitation Management (VRAM) programme.**”*** has been updated to “*To address the current healthcare education gap in agitation management, specifically in psychiatric settings, the education team at the Yong Loo Lin School of Medicine, National University of Singapore developed the Virtual Reality in Agitation Management (VRAM) programme.”*
- **Page 4, bottom**: The sentence *“Ethical approval was obtained from the [redacted for peer review], and informed consent was obtained from all participants prior to data collection.”* has been updated to *“Ethical approval was obtained from the National University of Singapore Institutional Review Board (NUS-IRB-2021-306), and informed consent was obtained from all participants prior to data collection.”*

These corrections do not affect the description, interpretation, or conclusions of the article. The authors apologize for any inconvenience caused.

