# Supplementary material for: Virtual reality for experiential learning: enhancing agitation management skills, confidence, and empathy in healthcare students
Source: Med Educ Online. 2025 Aug 6;30(1):2542809. doi: 10.1080/10872981.2025.2542809 (PMC12329851; doi:10.1080/10872981.2025.2542809)
Supplement: VRAM Manuscript_MEO_Supp Material_17 Jun.docx [file ZMEO_A_2542809_SM8070.docx]

**Using Virtual Reality for Experiential Learning: Enhancing Agitation Management Skills, Confidence, and Empathy in Healthcare Students**

**Supplementary Material**

**Annex A. Pre-MAGIC Questionnaire for Medical Students**

Please provide the last four digits of your mobile number for us to do matching of your responses.

|  |  |  |  |
| --- | --- | --- | --- |

**The following questions are on your personal experiences.**

Do you have experience in interacting with people with mental health conditions?

Yes

No

Have you ever encountered anyone who is agitated?

Yes

No

Do you have experience in managing someone who is agitated?

Yes

No

Have you ever encountered anyone who is depressed?

Yes

No

Do you have experience in managing someone who is depressed?

Yes

No

If given a choice, would you treat a patient with mental illness?

Yes

No

Does dealing with an angry patient remind you of an experience in the past?

Yes

No

How confident are you in managing patients who are agitated?

| 1  (Least) | 2 | 3 | 4 | 5 | 6 | 7  (Most) |
| --- | --- | --- | --- | --- | --- | --- |

How confident are you in using verbal de-escalation techniques to manage patients who are agitated?

| 1  (Least) | 2 | 3 | 4 | 5 | 6 | 7  (Most) |
| --- | --- | --- | --- | --- | --- | --- |

How confident are you in using chemical tranquilisation to manage patients who are agitated?

| 1  (Least) | 2 | 3 | 4 | 5 | 6 | 7  (Most) |
| --- | --- | --- | --- | --- | --- | --- |

How confident are you in using physical restraint to manage patients who are agitated?

| 1  (Least) | 2 | 3 | 4 | 5 | 6 | 7  (Most) |
| --- | --- | --- | --- | --- | --- | --- |

How confident are you in communicating with an agitated person?

| 1  (Least) | 2 | 3 | 4 | 5 | 6 | 7  (Most) |
| --- | --- | --- | --- | --- | --- | --- |

How confident are you in managing a situation (including the environment, other people etc) that involves an agitated patient?

| 1  (Least) | 2 | 3 | 4 | 5 | 6 | 7  (Most) |
| --- | --- | --- | --- | --- | --- | --- |

How would you rate your level of empathy towards patients?

| 1  (Least) | 2 | 3 | 4 | 5 | 6 | 7  (Most) |
| --- | --- | --- | --- | --- | --- | --- |

**The following questions assess attitudes and behavioural intentions towards people with mental illness. There is no correct answer. Please mark the box that best fits your opinion.**

I am more comfortable helping a person who has a physical illness than I am helping a person who has a mental illness.

| 1  (Strongly Disagree) | 2  (Disagree) | 3  (Neither Agree nor Disagree) | 4  (Agree) | 5  (Strongly Agree) |
| --- | --- | --- | --- | --- |

If a colleague with who I work with told me they had a mental illness, I would be just as willing to work with him / her.

| 1  (Strongly Disagree) | 2  (Disagree) | 3  (Neither Agree nor Disagree) | 4  (Agree) | 5  (Strongly Agree) |
| --- | --- | --- | --- | --- |

If I were under treatment for a mental illness, I would not disclose this to any of my colleagues.

| 1  (Strongly Disagree) | 2  (Disagree) | 3  (Neither Agree nor Disagree) | 4  (Agree) | 5  (Strongly Agree) |
| --- | --- | --- | --- | --- |

I would see myself as weak if I had a mental illness and could not fix it myself.

| 1  (Strongly Disagree) | 2  (Disagree) | 3  (Neither Agree nor Disagree) | 4  (Agree) | 5  (Strongly Agree) |
| --- | --- | --- | --- | --- |

I would be reluctant to seek help if I had a mental illness.

| 1  (Strongly Disagree) | 2  (Disagree) | 3  (Neither Agree nor Disagree) | 4  (Agree) | 5  (Strongly Agree) |
| --- | --- | --- | --- | --- |

Employers should hire a person with a managed mental illness if he / she is the best person for the job.

| 1  (Strongly Disagree) | 2  (Disagree) | 3  (Neither Agree nor Disagree) | 4  (Agree) | 5  (Strongly Agree) |
| --- | --- | --- | --- | --- |

I would still go to a physician if I knew that the physician had been treated for a mental illness.

| 1  (Strongly Disagree) | 2  (Disagree) | 3  (Neither Agree nor Disagree) | 4  (Agree) | 5  (Strongly Agree) |
| --- | --- | --- | --- | --- |

If I had a mental illness, I would tell my friends.

| 1  (Strongly Disagree) | 2  (Disagree) | 3  (Neither Agree nor Disagree) | 4  (Agree) | 5  (Strongly Agree) |
| --- | --- | --- | --- | --- |

Despite my professional beliefs, I have negative reactions towards people who have mental illness.

| 1  (Strongly Disagree) | 2  (Disagree) | 3  (Neither Agree nor Disagree) | 4  (Agree) | 5  (Strongly Agree) |
| --- | --- | --- | --- | --- |

There is little I can do to help people with mental illness.

| 1  (Strongly Disagree) | 2  (Disagree) | 3  (Neither Agree nor Disagree) | 4  (Agree) | 5  (Strongly Agree) |
| --- | --- | --- | --- | --- |

More than half of people with mental illness don’t try hard enough to get better.

| 1  (Strongly Disagree) | 2  (Disagree) | 3  (Neither Agree nor Disagree) | 4  (Agree) | 5  (Strongly Agree) |
| --- | --- | --- | --- | --- |

I would not want a person with a mental illness, even if it were appropriately managed, to work with children.

| 1  (Strongly Disagree) | 2  (Disagree) | 3  (Neither Agree nor Disagree) | 4  (Agree) | 5  (Strongly Agree) |
| --- | --- | --- | --- | --- |

Healthcare providers do not need to be advocates for people with mental illness.

| 1  (Strongly Disagree) | 2  (Disagree) | 3  (Neither Agree nor Disagree) | 4  (Agree) | 5  (Strongly Agree) |
| --- | --- | --- | --- | --- |

I would not mind if a person with a mental illness lived next door to me.

| 1  (Strongly Disagree) | 2  (Disagree) | 3  (Neither Agree nor Disagree) | 4  (Agree) | 5  (Strongly Agree) |
| --- | --- | --- | --- | --- |

I struggle to feel compassion for a person with mental illness.

| 1  (Strongly Disagree) | 2  (Disagree) | 3  (Neither Agree nor Disagree) | 4  (Agree) | 5  (Strongly Agree) |
| --- | --- | --- | --- | --- |

**The following questions are for purposes of gaining an understanding of your knowledge of various aspects of mental health. When responding, we are interested in your degree of knowledge, When choosing your response, consider that:**

Very unlikely = I am certain that it is NOT likely

Unlikely = I think it is unlikely but am not certain

Likely = I think it is likely but am not certain

Very likely = I am certain that it IS very likely

If someone became extremely nervous or anxious in one or more situations with other people (e.g., a party) or performance situations (e.g., presenting at a meeting) in which they were afraid of being evaluated by others and that they would act in a way that was humiliating or feel embarrassed, then to what extent do you think it is likely they have **Social Phobia:**

| 1  (Very unlikely) | 2  (Unlikely) | 3  (Likely) | 4  (Very likely) |
| --- | --- | --- | --- |

If someone experienced excessive worry about a number of events or activities where this level of concern was not warranted, had difficulty controlling this worry and had physical symptoms such as having tense muscles and feeling fatigued then to what extent do you think it is likely they have Generalised Anxiety Disorder:

| 1  (Very unlikely) | 2  (Unlikely) | 3  (Likely) | 4  (Very likely) |
| --- | --- | --- | --- |

If someone experienced a low mood for two or more weeks, had a loss of pleasure or interest in their normal activities and experienced changes in their appetite and sleep then to what extent do you think it is likely they have **Major Depressive Disorder:**

| 1  (Very unlikely) | 2  (Unlikely) | 3  (Likely) | 4  (Very likely) |
| --- | --- | --- | --- |

To what extent do you think it is likely that **Personality Disorders** are a category of mental illness:

| 1  (Very unlikely) | 2  (Unlikely) | 3  (Likely) | 4  (Very likely) |
| --- | --- | --- | --- |

To what extent do you think it is likely that **Dysthymia** is a disorder:

| 1  (Very unlikely) | 2  (Unlikely) | 3  (Likely) | 4  (Very likely) |
| --- | --- | --- | --- |

To what extent do you think it is likely that the diagnosis of **Agoraphobia** includes anxiety about situations where escape may be difficult or embarrassing:

| 1  (Very unlikely) | 2  (Unlikely) | 3  (Likely) | 4  (Very likely) |
| --- | --- | --- | --- |

To what extent do you think it is likely that the diagnosis of **Bipolar Disorder** includes experiencing periods of elevated (i.e., high) and periods of depressed (i.e., low) mood:

| 1  (Very unlikely) | 2  (Unlikely) | 3  (Likely) | 4  (Very likely) |
| --- | --- | --- | --- |

To what extent do you think it is likely that the diagnosis of **Drug Dependence** includes physical and psychological tolerance of the drug (i.e., require more of the drug to get the same effect):

| 1  (Very unlikely) | 2  (Unlikely) | 3  (Likely) | 4  (Very likely) |
| --- | --- | --- | --- |

To what extent do you think it is likely that in general in Singapore, **women are MORE likely to experience a mental illness of any kind compared to men:**

| 1  (Very unlikely) | 2  (Unlikely) | 3  (Likely) | 4  (Very likely) |
| --- | --- | --- | --- |

To what extent do you think it is likely that in general, in Singapore **men are MORE likely to experience an anxiety disorder compared to women:**

| 1  (Very unlikely) | 2  (Unlikely) | 3  (Likely) | 4  (Very likely) |
| --- | --- | --- | --- |

**When choosing your response, consider that:**

**Very unhelpful = I am certain that it is NOT helpful**

**Unhelpful = I think it is unhelpful but am not certain**

**Helpful = I think it is helpful but am not certain**

**Very helpful = I am certain it IS very helpful**

To what extent do you think it would be helpful for someone to **improve their quality of sleep** if they were having difficulties managing their emotions (e.g., becoming very anxious or depressed):

| 1  (Very unhelpful) | 2  (Unhelpful) | 3  (Helpful) | 4  (Very helpful) |
| --- | --- | --- | --- |

To what extent do you think it would be helpful for someone to **avoid all activities or situations that made them feel anxious** if they were having difficulties managing their emotions:

| 1  (Very unhelpful) | 2  (Unhelpful) | 3  (Helpful) | 4  (Very helpful) |
| --- | --- | --- | --- |

When choosing your response, consider that:

Very unlikely = I am certain that it is NOT likely

Unlikely = I think it is unlikely but am not certain

Likely = I think it is likely but am not certain

Very likely = I am certain that it IS very likely

To what extent do you think it is likely that **Cognitive Behaviour Therapy (CBT)** is a therapy based on challenging negative thoughts and increasing helpful behaviours

| 1  (Very unlikely) | 2  (Unlikely) | 3  (Likely) | 4  (Very likely) |
| --- | --- | --- | --- |

**Mental health professionals are bound by confidentiality. However, there are certain conditions under which this does not apply.**

**To what extent do you think it is likely that the following condition that would allow a mental health professional to break confidentiality:**

***If you are at immediate risk of harm to yourself or others***

| 1  (Very unlikely) | 2  (Unlikely) | 3  (Likely) | 4  (Very likely) |
| --- | --- | --- | --- |

**Mental health professionals are bound by confidentiality. However, there are certain conditions under which this does not apply.**

**To what extent do you think it is likely that the following condition that would allow a mental health professional to break confidentiality:**

***If your problem is not life-threatening and they want to assist others to better support you***

| 1  (Very unlikely) | 2  (Unlikely) | 3  (Likely) | 4  (Very likely) |
| --- | --- | --- | --- |

**Please indicate to what extent you agree with the following statements:**

|  | Strongly Disagree | Disagree | Neither agree or disagree | Agree | Strongly agree |
| --- | --- | --- | --- | --- | --- |
| I am confident that I know where to seek information about mental illnesses. |  |  |  |  |  |
| I am confident using the computer or telephone to seek information about mental illnesses. |  |  |  |  |  |
| I am confident attending face to face appointments to seek information about mental illness (e.g., seeing the GP). |  |  |  |  |  |
| I am confident I have access to resources (e.g., GP, internet, friends) that I can use to seek information about mental illness. |  |  |  |  |  |
| People with a mental illness could snap out if it if they wanted. |  |  |  |  |  |
| A mental illness is a sign of personal weakness. |  |  |  |  |  |
| A mental illness is not a real medical illness. |  |  |  |  |  |
| People with a mental illness are dangerous. |  |  |  |  |  |
| It is best to avoid people with a mental illness so that you don't develop this problem. |  |  |  |  |  |
| If I had a mental illness I would not tell anyone. |  |  |  |  |  |
| Seeing a mental health professional means you are not strong enough to manage your own difficulties. |  |  |  |  |  |
| If I had a mental illness, I would not seek help from a mental health professional. |  |  |  |  |  |
| I believe treatment for a mental illness, provided by a mental health professional, would not be effective. |  |  |  |  |  |

Please indicate to what extent you agree with the following statements:

|  | Definitely unwilling | Probably unwilling | Neither unwilling or willing | Probably willing | Definitely willing |
| --- | --- | --- | --- | --- | --- |
| How willing would you be to move next door to someone with a mental illness? |  |  |  |  |  |
| How willing would you be to spend an evening socialising with someone with a mental illness? |  |  |  |  |  |
| How willing would you be to make friends with someone with a mental illness? |  |  |  |  |  |
| How willing would you be to have someone with a mental illness start working closely with you on a job? |  |  |  |  |  |
| How willing would you be to have someone with a mental illness marry into your family? |  |  |  |  |  |
| How willing would you be to vote for a politician if you knew they had suffered a mental illness? |  |  |  |  |  |
| How willing would you be to employ someone if you knew they had a mental illness? |  |  |  |  |  |

**The following questions are on your personal attitudes about empathy in the context of health professions education and patient care.**

Physicians’ understanding of their patients’ feelings and the feelings of their patients’ families does not influence medical or surgical treatment.

| 1  (Strongly Disagree) | 2 | 3 | 4 | 5 | 6 | 7  (Strongly Agree) |
| --- | --- | --- | --- | --- | --- | --- |

Patients feel better when their physicians understand their feelings.

| 1  (Strongly Disagree) | 2 | 3 | 4 | 5 | 6 | 7  (Strongly Agree) |
| --- | --- | --- | --- | --- | --- | --- |

is difficult for a physician to view things from patients’ perspectives.

| 1  (Strongly Disagree) | 2 | 3 | 4 | 5 | 6 | 7  (Strongly Agree) |
| --- | --- | --- | --- | --- | --- | --- |

Understanding body language is as important as verbal communication in physician-patient relationships.

| 1  (Strongly Disagree) | 2 | 3 | 4 | 5 | 6 | 7  (Strongly Agree) |
| --- | --- | --- | --- | --- | --- | --- |

A physician’s sense of humour contributes to a better clinical outcome.

| 1  (Strongly Disagree) | 2 | 3 | 4 | 5 | 6 | 7  (Strongly Agree) |
| --- | --- | --- | --- | --- | --- | --- |

Because people are different, it is difficult to see things from patients’ perspectives.

| 1  (Strongly Disagree) | 2 | 3 | 4 | 5 | 6 | 7  (Strongly Agree) |
| --- | --- | --- | --- | --- | --- | --- |

Attention to patients’ emotions is not important in history taking.

| 1  (Strongly Disagree) | 2 | 3 | 4 | 5 | 6 | 7  (Strongly Agree) |
| --- | --- | --- | --- | --- | --- | --- |

Attentiveness to patients’ personal experiences does not influence treatment outcomes.

| 1  (Strongly Disagree) | 2 | 3 | 4 | 5 | 6 | 7  (Strongly Agree) |
| --- | --- | --- | --- | --- | --- | --- |

Physicians should try to stand in their patients’ shoes when providing care to them.

| 1  (Strongly Disagree) | 2 | 3 | 4 | 5 | 6 | 7  (Strongly Agree) |
| --- | --- | --- | --- | --- | --- | --- |

Patients value a physician’s understanding of their feelings which is therapeutic in its own right.

| 1  (Strongly Disagree) | 2 | 3 | 4 | 5 | 6 | 7  (Strongly Agree) |
| --- | --- | --- | --- | --- | --- | --- |

Patients’ illnesses can be cured only by medical or surgical treatment; therefore, physicians’ emotional ties with their patients do not have a significant influence in medical or surgical treatment.

| 1  (Strongly Disagree) | 2 | 3 | 4 | 5 | 6 | 7  (Strongly Agree) |
| --- | --- | --- | --- | --- | --- | --- |

Asking patients about what is happening in their personal lives is not helpful in understanding their physical complaints.

| 1  (Strongly Disagree) | 2 | 3 | 4 | 5 | 6 | 7  (Strongly Agree) |
| --- | --- | --- | --- | --- | --- | --- |

Physicians should try to understand what is going on in their patients’ minds by paying attention to their non-verbal cues and body language.

| 1  (Strongly Disagree) | 2 | 3 | 4 | 5 | 6 | 7  (Strongly Agree) |
| --- | --- | --- | --- | --- | --- | --- |

I believe that emotion has no place in the treatment of medical illness.

| 1  (Strongly Disagree) | 2 | 3 | 4 | 5 | 6 | 7  (Strongly Agree) |
| --- | --- | --- | --- | --- | --- | --- |

Empathy is a therapeutic skill without which the physician’s success is limited.

| 1  (Strongly Disagree) | 2 | 3 | 4 | 5 | 6 | 7  (Strongly Agree) |
| --- | --- | --- | --- | --- | --- | --- |

Physicians’ understanding of the emotional status of their parents, as well as that of their families is one important component of the physician-patient relationship.

| 1  (Strongly Disagree) | 2 | 3 | 4 | 5 | 6 | 7  (Strongly Agree) |
| --- | --- | --- | --- | --- | --- | --- |

Physicians should try to think like their patients in order to render better care.

| 1  (Strongly Disagree) | 2 | 3 | 4 | 5 | 6 | 7  (Strongly Agree) |
| --- | --- | --- | --- | --- | --- | --- |

Physicians should not allow themselves to be influenced by strong personal bonds between their patients and their family members.

| 1  (Strongly Disagree) | 2 | 3 | 4 | 5 | 6 | 7  (Strongly Agree) |
| --- | --- | --- | --- | --- | --- | --- |

I do not enjoy reading non-medical literature or the arts.

| 1  (Strongly Disagree) | 2 | 3 | 4 | 5 | 6 | 7  (Strongly Agree) |
| --- | --- | --- | --- | --- | --- | --- |

I believe that empathy is an important therapeutic factor in medical treatment.

| 1  (Strongly Disagree) | 2 | 3 | 4 | 5 | 6 | 7  (Strongly Agree) |
| --- | --- | --- | --- | --- | --- | --- |

**Annex B. Post-MAGIC Questionnaire for Medical Students**

Please provide the last four digits of your mobile number for us to do matching of your responses.

|  |  |  |  |
| --- | --- | --- | --- |

**The following questions are on your personal experiences with agitated patients.**

If given a choice, would you treat a patient with mental illness?

Yes

No

How confident are you in managing patients who are agitated?

| 1  (Least) | 2 | 3 | 4 | 5 | 6 | 7  (Most) |
| --- | --- | --- | --- | --- | --- | --- |

How confident are you in using verbal de-escalation techniques to manage patients who are agitated?

| 1  (Least) | 2 | 3 | 4 | 5 | 6 | 7  (Most) |
| --- | --- | --- | --- | --- | --- | --- |

How confident are you in using chemical tranquilisation to manage patients who are agitated?

| 1  (Least) | 2 | 3 | 4 | 5 | 6 | 7  (Most) |
| --- | --- | --- | --- | --- | --- | --- |

How confident are you in using physical restraint to manage patients who are agitated?

| 1  (Least) | 2 | 3 | 4 | 5 | 6 | 7  (Most) |
| --- | --- | --- | --- | --- | --- | --- |

How confident are you in communicating with an agitated person?

| 1  (Least) | 2 | 3 | 4 | 5 | 6 | 7  (Most) |
| --- | --- | --- | --- | --- | --- | --- |

How confident are you in managing a situation (including the environment, other people etc) that involves an agitated patient?

| 1  (Least) | 2 | 3 | 4 | 5 | 6 | 7  (Most) |
| --- | --- | --- | --- | --- | --- | --- |

How would you rate your level of empathy towards patients?

| 1  (Least) | 2 | 3 | 4 | 5 | 6 | 7  (Most) |
| --- | --- | --- | --- | --- | --- | --- |

I am ready to provide care for an agitated patient.

| 1  (Least) | 2 | 3 | 4 | 5 | 6 | 7  (Most) |
| --- | --- | --- | --- | --- | --- | --- |

I am competent in caring for patients with mental health conditions.

| 1  (Least) | 2 | 3 | 4 | 5 | 6 | 7  (Most) |
| --- | --- | --- | --- | --- | --- | --- |

I am competent in managing agitated patients.

| 1  (Least) | 2 | 3 | 4 | 5 | 6 | 7  (Most) |
| --- | --- | --- | --- | --- | --- | --- |

**The following questions attitudes and behavioural intentions towards people with mental illness. There is no correct answer. Please mark the box that best fits your opinion.**

I am more comfortable helping a person who has a physical illness than I am helping a person who has a mental illness.

| 1  (Strongly Disagree) | 2  (Disagree) | 3  (Neither Agree nor Disagree) | 4  (Agree) | 5  (Strongly Agree) |
| --- | --- | --- | --- | --- |

If a colleague with who I work with told me they had a mental illness, I would be just as willing to work with him / her.

| 1  (Strongly Disagree) | 2  (Disagree) | 3  (Neither Agree nor Disagree) | 4  (Agree) | 5  (Strongly Agree) |
| --- | --- | --- | --- | --- |

If I were under treatment for a mental illness, I would not disclose this to any of my colleagues.

| 1  (Strongly Disagree) | 2  (Disagree) | 3  (Neither Agree nor Disagree) | 4  (Agree) | 5  (Strongly Agree) |
| --- | --- | --- | --- | --- |

I would see myself as weak if I had a mental illness and could not fix it myself.

| 1  (Strongly Disagree) | 2  (Disagree) | 3  (Neither Agree nor Disagree) | 4  (Agree) | 5  (Strongly Agree) |
| --- | --- | --- | --- | --- |

I would be reluctant to seek help if I had a mental illness.

| 1  (Strongly Disagree) | 2  (Disagree) | 3  (Neither Agree nor Disagree) | 4  (Agree) | 5  (Strongly Agree) |
| --- | --- | --- | --- | --- |

Employers should hire a person with a managed mental illness if he / she is the best person for the job.

| 1  (Strongly Disagree) | 2  (Disagree) | 3  (Neither Agree nor Disagree) | 4  (Agree) | 5  (Strongly Agree) |
| --- | --- | --- | --- | --- |

I would still go to a physician if I knew that the physician had been treated for a mental illness.

| 1  (Strongly Disagree) | 2  (Disagree) | 3  (Neither Agree nor Disagree) | 4  (Agree) | 5  (Strongly Agree) |
| --- | --- | --- | --- | --- |

If I had a mental illness, I would tell my friends.

| 1  (Strongly Disagree) | 2  (Disagree) | 3  (Neither Agree nor Disagree) | 4  (Agree) | 5  (Strongly Agree) |
| --- | --- | --- | --- | --- |

Despite my professional beliefs, I have negative reactions towards people who have mental illness.

| 1  (Strongly Disagree) | 2  (Disagree) | 3  (Neither Agree nor Disagree) | 4  (Agree) | 5  (Strongly Agree) |
| --- | --- | --- | --- | --- |

There is little I can do to help people with mental illness.

| 1  (Strongly Disagree) | 2  (Disagree) | 3  (Neither Agree nor Disagree) | 4  (Agree) | 5  (Strongly Agree) |
| --- | --- | --- | --- | --- |

More than half of people with mental illness don’t try hard enough to get better.

| 1  (Strongly Disagree) | 2  (Disagree) | 3  (Neither Agree nor Disagree) | 4  (Agree) | 5  (Strongly Agree) |
| --- | --- | --- | --- | --- |

I would not want a person with a mental illness, even if it were appropriately managed, to work with children.

| 1  (Strongly Disagree) | 2  (Disagree) | 3  (Neither Agree nor Disagree) | 4  (Agree) | 5  (Strongly Agree) |
| --- | --- | --- | --- | --- |

Healthcare providers do not need to be advocates for people with mental illness.

| 1  (Strongly Disagree) | 2  (Disagree) | 3  (Neither Agree nor Disagree) | 4  (Agree) | 5  (Strongly Agree) |
| --- | --- | --- | --- | --- |

I would not mind if a person with a mental illness lived next door to me.

| 1  (Strongly Disagree) | 2  (Disagree) | 3  (Neither Agree nor Disagree) | 4  (Agree) | 5  (Strongly Agree) |
| --- | --- | --- | --- | --- |

I struggle to feel compassion for a person with mental illness.

| 1  (Strongly Disagree) | 2  (Disagree) | 3  (Neither Agree nor Disagree) | 4  (Agree) | 5  (Strongly Agree) |
| --- | --- | --- | --- | --- |

**The following questions are for purposes of gaining an understanding of your knowledge of various aspects of mental health. When responding, we are interested in your degree of knowledge, When choosing your response, consider that:**

Very unlikely = I am certain that it is NOT likely

Unlikely = I think it is unlikely but am not certain

Likely = I think it is likely but am not certain

Very likely = I am certain that it IS very likely

If someone became extremely nervous or anxious in one or more situations with other people (e.g., a party) or performance situations (e.g., presenting at a meeting) in which they were afraid of being evaluated by others and that they would act in a way that was humiliating or feel embarrassed, then to what extent do you think it is likely they have **Social Phobia:**

| 1  (Very unlikely) | 2  (Unlikely) | 3  (Likely) | 4  (Very likely) |
| --- | --- | --- | --- |

If someone experienced excessive worry about a number of events or activities where this level of concern was not warranted, had difficulty controlling this worry and had physical symptoms such as having tense muscles and feeling fatigued then to what extent do you think it is likely they have Generalised Anxiety Disorder:

| 1  (Very unlikely) | 2  (Unlikely) | 3  (Likely) | 4  (Very likely) |
| --- | --- | --- | --- |

If someone experienced a low mood for two or more weeks, had a loss of pleasure or interest in their normal activities and experienced changes in their appetite and sleep then to what extent do you think it is likely they have **Major Depressive Disorder:**

| 1  (Very unlikely) | 2  (Unlikely) | 3  (Likely) | 4  (Very likely) |
| --- | --- | --- | --- |

To what extent do you think it is likely that **Personality Disorders** are a category of mental illness:

| 1  (Very unlikely) | 2  (Unlikely) | 3  (Likely) | 4  (Very likely) |
| --- | --- | --- | --- |

To what extent do you think it is likely that **Dysthymia** is a disorder:

| 1  (Very unlikely) | 2  (Unlikely) | 3  (Likely) | 4  (Very likely) |
| --- | --- | --- | --- |

To what extent do you think it is likely that the diagnosis of **Agoraphobia** includes anxiety about situations where escape may be difficult or embarrassing:

| 1  (Very unlikely) | 2  (Unlikely) | 3  (Likely) | 4  (Very likely) |
| --- | --- | --- | --- |

To what extent do you think it is likely that the diagnosis of **Bipolar Disorder** includes experiencing periods of elevated (i.e., high) and periods of depressed (i.e., low) mood:

| 1  (Very unlikely) | 2  (Unlikely) | 3  (Likely) | 4  (Very likely) |
| --- | --- | --- | --- |

To what extent do you think it is likely that the diagnosis of **Drug Dependence** includes physical and psychological tolerance of the drug (i.e., require more of the drug to get the same effect):

| 1  (Very unlikely) | 2  (Unlikely) | 3  (Likely) | 4  (Very likely) |
| --- | --- | --- | --- |

To what extent do you think it is likely that in general in Singapore, **women are MORE likely to experience a mental illness of any kind compared to men:**

| 1  (Very unlikely) | 2  (Unlikely) | 3  (Likely) | 4  (Very likely) |
| --- | --- | --- | --- |

To what extent do you think it is likely that in general, in Singapore **men are MORE likely to experience an anxiety disorder compared to women:**

| 1  (Very unlikely) | 2  (Unlikely) | 3  (Likely) | 4  (Very likely) |
| --- | --- | --- | --- |

**When choosing your response, consider that:**

**Very unhelpful = I am certain that it is NOT helpful**

**Unhelpful = I think it is unhelpful but am not certain**

**Helpful = I think it is helpful but am not certain**

**Very helpful = I am certain it IS very helpful**

To what extent do you think it would be helpful for someone to **improve their quality of sleep** if they were having difficulties managing their emotions (e.g., becoming very anxious or depressed):

| 1  (Very unhelpful) | 2  (Unhelpful) | 3  (Helpful) | 4  (Very helpful) |
| --- | --- | --- | --- |

To what extent do you think it would be helpful for someone to **avoid all activities or situations that made them feel anxious** if they were having difficulties managing their emotions:

| 1  (Very unhelpful) | 2  (Unhelpful) | 3  (Helpful) | 4  (Very helpful) |
| --- | --- | --- | --- |

When choosing your response, consider that:

Very unlikely = I am certain that it is NOT likely

Unlikely = I think it is unlikely but am not certain

Likely = I think it is likely but am not certain

Very likely = I am certain that it IS very likely

To what extent do you think it is likely that **Cognitive Behaviour Therapy (CBT)** is a therapy based on challenging negative thoughts and increasing helpful behaviours

| 1  (Very unlikely) | 2  (Unlikely) | 3  (Likely) | 4  (Very likely) |
| --- | --- | --- | --- |

**Mental health professionals are bound by confidentiality. However, there are certain conditions under which this does not apply.**

**To what extent do you think it is likely that the following condition that would allow a mental health professional to break confidentiality:**

***If you are at immediate risk of harm to yourself or others***

| 1  (Very unlikely) | 2  (Unlikely) | 3  (Likely) | 4  (Very likely) |
| --- | --- | --- | --- |

**Mental health professionals are bound by confidentiality. However, there are certain conditions under which this does not apply.**

**To what extent do you think it is likely that the following condition that would allow a mental health professional to break confidentiality:**

***If your problem is not life-threatening and they want to assist others to better support you***

| 1  (Very unlikely) | 2  (Unlikely) | 3  (Likely) | 4  (Very likely) |
| --- | --- | --- | --- |

**Please indicate to what extent you agree with the following statements:**

|  | Strongly Disagree | Disagree | Neither agree or disagree | Agree | Strongly agree |
| --- | --- | --- | --- | --- | --- |
| I am confident that I know where to seek information about mental illnesses. |  |  |  |  |  |
| I am confident using the computer or telephone to seek information about mental illnesses. |  |  |  |  |  |
| I am confident attending face to face appointments to seek information about mental illness (e.g., seeing the GP). |  |  |  |  |  |
| I am confident I have access to resources (e.g., GP, internet, friends) that I can use to seek information about mental illness. |  |  |  |  |  |
| People with a mental illness could snap out if it if they wanted. |  |  |  |  |  |
| A mental illness is a sign of personal weakness. |  |  |  |  |  |
| A mental illness is not a real medical illness. |  |  |  |  |  |
| People with a mental illness are dangerous. |  |  |  |  |  |
| It is best to avoid people with a mental illness so that you don't develop this problem. |  |  |  |  |  |
| If I had a mental illness I would not tell anyone. |  |  |  |  |  |
| Seeing a mental health professional means you are not strong enough to manage your own difficulties. |  |  |  |  |  |
| If I had a mental illness, I would not seek help from a mental health professional. |  |  |  |  |  |
| I believe treatment for a mental illness, provided by a mental health professional, would not be effective. |  |  |  |  |  |

Please indicate to what extent you agree with the following statements:

|  | Definitely unwilling | Probably unwilling | Neither unwilling or willing | Probably willing | Definitely willing |
| --- | --- | --- | --- | --- | --- |
| How willing would you be to move next door to someone with a mental illness? |  |  |  |  |  |
| How willing would you be to spend an evening socialising with someone with a mental illness? |  |  |  |  |  |
| How willing would you be to make friends with someone with a mental illness? |  |  |  |  |  |
| How willing would you be to have someone with a mental illness start working closely with you on a job? |  |  |  |  |  |
| How willing would you be to have someone with a mental illness marry into your family? |  |  |  |  |  |
| How willing would you be to vote for a politician if you knew they had suffered a mental illness? |  |  |  |  |  |
| How willing would you be to employ someone if you knew they had a mental illness? |  |  |  |  |  |

**The following questions are on your personal attitudes about empathy in the context of health professions education and patient care.**

Health care providers’ understanding of their patients’ feelings and the feelings of their patients’ families does not influence medical or surgical treatment

| 1  (Strongly Disagree) | 2 | 3 | 4 | 5 | 6 | 7  (Strongly Agree) |
| --- | --- | --- | --- | --- | --- | --- |

Patients feel better when their physicians understand their feelings.

| 1  (Strongly Disagree) | 2 | 3 | 4 | 5 | 6 | 7  (Strongly Agree) |
| --- | --- | --- | --- | --- | --- | --- |

It is difficult for a physician to view things from patients’ perspectives.

| 1  (Strongly Disagree) | 2 | 3 | 4 | 5 | 6 | 7  (Strongly Agree) |
| --- | --- | --- | --- | --- | --- | --- |

Understanding body language is as important as verbal communication in physician-patient relationships.

| 1  (Strongly Disagree) | 2 | 3 | 4 | 5 | 6 | 7  (Strongly Agree) |
| --- | --- | --- | --- | --- | --- | --- |

A physician’s sense of humour contributes to a better clinical outcome.

| 1  (Strongly Disagree) | 2 | 3 | 4 | 5 | 6 | 7  (Strongly Agree) |
| --- | --- | --- | --- | --- | --- | --- |

Because people are different, it is difficult to see things from patients’ perspectives.

| 1  (Strongly Disagree) | 2 | 3 | 4 | 5 | 6 | 7  (Strongly Agree) |
| --- | --- | --- | --- | --- | --- | --- |

Attention to patients’ emotions is not important in history taking.

| 1  (Strongly Disagree) | 2 | 3 | 4 | 5 | 6 | 7  (Strongly Agree) |
| --- | --- | --- | --- | --- | --- | --- |

Attentiveness to patients’ personal experiences does not influence treatment outcomes.

| 1  (Strongly Disagree) | 2 | 3 | 4 | 5 | 6 | 7  (Strongly Agree) |
| --- | --- | --- | --- | --- | --- | --- |

Physicians should try to stand in their patients’ shoes when providing care to them.

| 1  (Strongly Disagree) | 2 | 3 | 4 | 5 | 6 | 7  (Strongly Agree) |
| --- | --- | --- | --- | --- | --- | --- |

Patients value a physician’s understanding of their feelings which is therapeutic in its own right.

| 1  (Strongly Disagree) | 2 | 3 | 4 | 5 | 6 | 7  (Strongly Agree) |
| --- | --- | --- | --- | --- | --- | --- |

Patients’ illnesses can be cured only by medical or surgical treatment; therefore, physicians’ emotional ties with their patients do not have a significant influence in medical or surgical treatment.

| 1  (Strongly Disagree) | 2 | 3 | 4 | 5 | 6 | 7  (Strongly Agree) |
| --- | --- | --- | --- | --- | --- | --- |

Asking patients about what is happening in their personal lives is not helpful in understanding their physical complaints.

| 1  (Strongly Disagree) | 2 | 3 | 4 | 5 | 6 | 7  (Strongly Agree) |
| --- | --- | --- | --- | --- | --- | --- |

Physicians should try to understand what is going on in their patients’ minds by paying attention to their non-verbal cues and body language.

| 1  (Strongly Disagree) | 2 | 3 | 4 | 5 | 6 | 7  (Strongly Agree) |
| --- | --- | --- | --- | --- | --- | --- |

I believe that emotion has no place in the treatment of medical illness.

| 1  (Strongly Disagree) | 2 | 3 | 4 | 5 | 6 | 7  (Strongly Agree) |
| --- | --- | --- | --- | --- | --- | --- |

Empathy is a therapeutic skill without which the physician’s success is limited.

| 1  (Strongly Disagree) | 2 | 3 | 4 | 5 | 6 | 7  (Strongly Agree) |
| --- | --- | --- | --- | --- | --- | --- |

Physicians’ understanding of the emotional status of their parents, as well as that of their families is one important component of the physician-patient relationship.

| 1  (Strongly Disagree) | 2 | 3 | 4 | 5 | 6 | 7  (Strongly Agree) |
| --- | --- | --- | --- | --- | --- | --- |

Physicians should try to think like their patients in order to render better care.

| 1  (Strongly Disagree) | 2 | 3 | 4 | 5 | 6 | 7  (Strongly Agree) |
| --- | --- | --- | --- | --- | --- | --- |

Physicians should not allow themselves to be influenced by strong personal bonds between their patients and their family members.

| 1  (Strongly Disagree) | 2 | 3 | 4 | 5 | 6 | 7  (Strongly Agree) |
| --- | --- | --- | --- | --- | --- | --- |

I do not enjoy reading non-medical literature or the arts.

| 1  (Strongly Disagree) | 2 | 3 | 4 | 5 | 6 | 7  (Strongly Agree) |
| --- | --- | --- | --- | --- | --- | --- |

I believe that empathy is an important therapeutic factor in medical treatment.

| 1  (Strongly Disagree) | 2 | 3 | 4 | 5 | 6 | 7  (Strongly Agree) |
| --- | --- | --- | --- | --- | --- | --- |

**The following questions are on your experience with using the VR software.**

What is the level of immersion you experienced?

| 1  (Extremely Low) | 2  (Very Low) | 3  (Low) | 4  (Neutral) | 5  (High) | 6  (Very High) | 7  (Extremely High) |
| --- | --- | --- | --- | --- | --- | --- |

Any additional comments and / or suggestions relevant to the question above:

|  |
| --- |

What was your level of enjoyment of the VR experience?

| 1  (Extremely Low) | 2  (Very Low) | 3  (Low) | 4  (Neutral) | 5  (High) | 6  (Very High) | 7  (Extremely High) |
| --- | --- | --- | --- | --- | --- | --- |

Any additional comments and / or suggestions relevant to the question above:

|  |
| --- |

How was the quality of the graphics?

| 1  (Extremely Low) | 2  (Very Low) | 3  (Low) | 4  (Neutral) | 5  (High) | 6  (Very High) | 7  (Extremely High) |
| --- | --- | --- | --- | --- | --- | --- |

Any additional comments and / or suggestions relevant to the question above:

|  |
| --- |

How was the quality of sound?

| 1  (Extremely Low) | 2  (Very Low) | 3  (Low) | 4  (Neutral) | 5  (High) | 6  (Very High) | 7  (Extremely High) |
| --- | --- | --- | --- | --- | --- | --- |

Any additional comments and / or suggestions relevant to the question above:

|  |
| --- |

How was the quality of the VR technology overall (i.e., hardware and peripherals)?

| 1  (Extremely Low) | 2  (Very Low) | 3  (Low) | 4  (Neutral) | 5  (High) | 6  (Very High) | 7  (Extremely High) |
| --- | --- | --- | --- | --- | --- | --- |

Any additional comments and / or suggestions relevant to the question above:

|  |
| --- |

How easy was it to use the navigation system (e.g., teleportation) in the virtual environment?

| 1  (Extremely Low) | 2  (Very Low) | 3  (Low) | 4  (Neutral) | 5  (High) | 6  (Very High) | 7  (Extremely High) |
| --- | --- | --- | --- | --- | --- | --- |

Any additional comments and / or suggestions relevant to the question above:

|  |
| --- |

How easy was it to physically move in the virtual environment?

| 1  (Extremely Low) | 2  (Very Low) | 3  (Low) | 4  (Neutral) | 5  (High) | 6  (Very High) | 7  (Extremely High) |
| --- | --- | --- | --- | --- | --- | --- |

Any additional comments and / or suggestions relevant to the question above:

|  |
| --- |

How easy was it to pick up and / or place items in the virtual environment?

| 1  (Extremely Low) | 2  (Very Low) | 3  (Low) | 4  (Neutral) | 5  (High) | 6  (Very High) | 7  (Extremely High) |
| --- | --- | --- | --- | --- | --- | --- |

Any additional comments and / or suggestions relevant to the question above:

|  |
| --- |

How easy was it to use items in the virtual environment?

| 1  (Extremely Low) | 2  (Very Low) | 3  (Low) | 4  (Neutral) | 5  (High) | 6  (Very High) | 7  (Extremely High) |
| --- | --- | --- | --- | --- | --- | --- |

Any additional comments and / or suggestions relevant to the question above:

|  |
| --- |

How easy was the two-handed interaction e.g., grab the tablet with one hand, and push the button with the other hand?

| 1  (Extremely Low) | 2  (Very Low) | 3  (Low) | 4  (Neutral) | 5  (High) | 6  (Very High) | 7  (Extremely High) |
| --- | --- | --- | --- | --- | --- | --- |

Any additional comments and / or suggestions relevant to the question above:

|  |
| --- |

How easy was it to complete the tutorial?

| 1  (Extremely Low) | 2  (Very Low) | 3  (Low) | 4  (Neutral) | 5  (High) | 6  (Very High) | 7  (Extremely High) |
| --- | --- | --- | --- | --- | --- | --- |

Any additional comments and / or suggestions relevant to the question above:

|  |
| --- |

How helpful was / were the tutorial(s)?

| 1  (Extremely Low) | 2  (Very Low) | 3  (Low) | 4  (Neutral) | 5  (High) | 6  (Very High) | 7  (Extremely High) |
| --- | --- | --- | --- | --- | --- | --- |

Any additional comments and / or suggestions relevant to the question above:

|  |
| --- |

How did you feel about the duration of the tutorial?

| 1  (Extremely Low) | 2  (Very Low) | 3  (Low) | 4  (Neutral) | 5  (High) | 6  (Very High) | 7  (Extremely High) |
| --- | --- | --- | --- | --- | --- | --- |

Any additional comments and / or suggestions relevant to the question above:

|  |
| --- |

How helpful were the in-game instructions for the task you needed to perform?

| 1  (Extremely Low) | 2  (Very Low) | 3  (Low) | 4  (Neutral) | 5  (High) | 6  (Very High) | 7  (Extremely High) |
| --- | --- | --- | --- | --- | --- | --- |

Any additional comments and / or suggestions relevant to the question above:

|  |
| --- |

How helpful were the in-game prompts e.g., arrows showing the direction or labels?

| 1  (Extremely Low) | 2  (Very Low) | 3  (Low) | 4  (Neutral) | 5  (High) | 6  (Very High) | 7  (Extremely High) |
| --- | --- | --- | --- | --- | --- | --- |

Any additional comments and / or suggestions relevant to the question above:

|  |
| --- |

Did you experience nausea?

| 1  (Extremely Low) | 2  (Very Low) | 3  (Low) | 4  (Neutral) | 5  (High) | 6  (Very High) | 7  (Extremely High) |
| --- | --- | --- | --- | --- | --- | --- |

Any additional comments and / or suggestions relevant to the question above:

|  |
| --- |

Did you experience disorientation?

| 1  (Extremely Low) | 2  (Very Low) | 3  (Low) | 4  (Neutral) | 5  (High) | 6  (Very High) | 7  (Extremely High) |
| --- | --- | --- | --- | --- | --- | --- |

Any additional comments and / or suggestions relevant to the question above:

|  |
| --- |

Did you experience dizziness?

| 1  (Extremely Low) | 2  (Very Low) | 3  (Low) | 4  (Neutral) | 5  (High) | 6  (Very High) | 7  (Extremely High) |
| --- | --- | --- | --- | --- | --- | --- |

Any additional comments and / or suggestions relevant to the question above:

|  |
| --- |

Did you experience fatigue?

| 1  (Extremely Low) | 2  (Very Low) | 3  (Low) | 4  (Neutral) | 5  (High) | 6  (Very High) | 7  (Extremely High) |
| --- | --- | --- | --- | --- | --- | --- |

Any additional comments and / or suggestions relevant to the question above:

|  |
| --- |

Did you experience instability?

| 1  (Extremely Low) | 2  (Very Low) | 3  (Low) | 4  (Neutral) | 5  (High) | 6  (Very High) | 7  (Extremely High) |
| --- | --- | --- | --- | --- | --- | --- |

Any additional comments and / or suggestions relevant to the question above:

|  |
| --- |

**The following questions are on your experiences during the session.**

The VR software helped me understand symptoms faced by a patient with mental health conditions in a realistic manner.

| 1  (Strongly Disagree) | 2 | 3 | 4 | 5 | 6 | 7  (Strongly Agree) |
| --- | --- | --- | --- | --- | --- | --- |

The scenarios depicted are realistic and have learning values.

| 1  (Strongly Disagree) | 2 | 3 | 4 | 5 | 6 | 7  (Strongly Agree) |
| --- | --- | --- | --- | --- | --- | --- |

The VR software provided an immersive experience.

| 1  (Strongly Disagree) | 2 | 3 | 4 | 5 | 6 | 7  (Strongly Agree) |
| --- | --- | --- | --- | --- | --- | --- |

I would like to recommend other people to go through this experience because I find it helpful.

| 1  (Strongly Disagree) | 2 | 3 | 4 | 5 | 6 | 7  (Strongly Agree) |
| --- | --- | --- | --- | --- | --- | --- |

The session was conducted in an engaging manner.

| 1  (Strongly Disagree) | 2 | 3 | 4 | 5 | 6 | 7  (Strongly Agree) |
| --- | --- | --- | --- | --- | --- | --- |

The debrief session was useful for consolidating my learning.

| 1  (Strongly Disagree) | 2 | 3 | 4 | 5 | 6 | 7  (Strongly Agree) |
| --- | --- | --- | --- | --- | --- | --- |

Why was the debrief session useful / not useful?

|  |
| --- |

I would like to use the VR software again.

| 1  (Strongly Disagree) | 2 | 3 | 4 | 5 | 6 | 7  (Strongly Agree) |
| --- | --- | --- | --- | --- | --- | --- |

I now have a better understanding of how to manage agitated patients.

| 1  (Strongly Disagree) | 2 | 3 | 4 | 5 | 6 | 7  (Strongly Agree) |
| --- | --- | --- | --- | --- | --- | --- |

The VR software allowed me to learn how to manage agitated patients and psychiatric patients more efficiently than just attending didactic lectures and tutorials.

| 1  (Strongly Disagree) | 2 | 3 | 4 | 5 | 6 | 7  (Strongly Agree) |
| --- | --- | --- | --- | --- | --- | --- |

Please highlight the strengths / weaknesses of this session.

|  |
| --- |

Suggestions on how to improve this session.

|  |
| --- |

What other scenarios would you like to experience through the use of VR software?

|  |
| --- |

Any other comments?

|  |
| --- |

**Annex C. Pre-MAGIC Questionnaire for Nursing Students**

Please provide the last four digits of your mobile number for us to do matching of your responses.

|  |  |  |  |
| --- | --- | --- | --- |

**The following questions are on your personal experiences.**

Do you have experience in interacting with people with mental health conditions?

Yes

No

Have you ever encountered anyone who is agitated?

Yes

No

Do you have experience in managing someone who is agitated?

Yes

No

Have you ever encountered anyone who is depressed?

Yes

No

Do you have experience in managing someone who is depressed?

Yes

No

If given a choice, would you treat a patient with mental illness?

Yes

No

Does dealing with an angry patient remind you of an experience in the past?

Yes

No

How confident are you in managing patients who are agitated?

| 1  (Least) | 2 | 3 | 4 | 5 | 6 | 7  (Most) |
| --- | --- | --- | --- | --- | --- | --- |

How confident are you in using verbal de-escalation techniques to manage patients who are agitated?

| 1  (Least) | 2 | 3 | 4 | 5 | 6 | 7  (Most) |
| --- | --- | --- | --- | --- | --- | --- |

How confident are you in using chemical tranquilisation to manage patients who are agitated?

| 1  (Least) | 2 | 3 | 4 | 5 | 6 | 7  (Most) |
| --- | --- | --- | --- | --- | --- | --- |

How confident are you in using physical restraint to manage patients who are agitated?

| 1  (Least) | 2 | 3 | 4 | 5 | 6 | 7  (Most) |
| --- | --- | --- | --- | --- | --- | --- |

How confident are you in communicating with an agitated person?

| 1  (Least) | 2 | 3 | 4 | 5 | 6 | 7  (Most) |
| --- | --- | --- | --- | --- | --- | --- |

How confident are you in managing a situation (including the environment, other people etc) that involves an agitated patient?

| 1  (Least) | 2 | 3 | 4 | 5 | 6 | 7  (Most) |
| --- | --- | --- | --- | --- | --- | --- |

How would you rate your level of empathy towards patients?

| 1  (Least) | 2 | 3 | 4 | 5 | 6 | 7  (Most) |
| --- | --- | --- | --- | --- | --- | --- |

**The following questions assess attitudes and behavioural intentions towards people with mental illness. There is no correct answer. Please mark the box that best fits your opinion.**

I am more comfortable helping a person who has a physical illness than I am helping a person who has a mental illness.

| 1  (Strongly Disagree) | 2  (Disagree) | 3  (Neither Agree nor Disagree) | 4  (Agree) | 5  (Strongly Agree) |
| --- | --- | --- | --- | --- |

If a colleague with who I work with told me they had a mental illness, I would be just as willing to work with him / her.

| 1  (Strongly Disagree) | 2  (Disagree) | 3  (Neither Agree nor Disagree) | 4  (Agree) | 5  (Strongly Agree) |
| --- | --- | --- | --- | --- |

If I were under treatment for a mental illness, I would not disclose this to any of my colleagues.

| 1  (Strongly Disagree) | 2  (Disagree) | 3  (Neither Agree nor Disagree) | 4  (Agree) | 5  (Strongly Agree) |
| --- | --- | --- | --- | --- |

I would see myself as weak if I had a mental illness and could not fix it myself.

| 1  (Strongly Disagree) | 2  (Disagree) | 3  (Neither Agree nor Disagree) | 4  (Agree) | 5  (Strongly Agree) |
| --- | --- | --- | --- | --- |

I would be reluctant to seek help if I had a mental illness.

| 1  (Strongly Disagree) | 2  (Disagree) | 3  (Neither Agree nor Disagree) | 4  (Agree) | 5  (Strongly Agree) |
| --- | --- | --- | --- | --- |

Employers should hire a person with a managed mental illness if he / she is the best person for the job.

| 1  (Strongly Disagree) | 2  (Disagree) | 3  (Neither Agree nor Disagree) | 4  (Agree) | 5  (Strongly Agree) |
| --- | --- | --- | --- | --- |

I would still go to a physician if I knew that the physician had been treated for a mental illness.

| 1  (Strongly Disagree) | 2  (Disagree) | 3  (Neither Agree nor Disagree) | 4  (Agree) | 5  (Strongly Agree) |
| --- | --- | --- | --- | --- |

If I had a mental illness, I would tell my friends.

| 1  (Strongly Disagree) | 2  (Disagree) | 3  (Neither Agree nor Disagree) | 4  (Agree) | 5  (Strongly Agree) |
| --- | --- | --- | --- | --- |

Despite my professional beliefs, I have negative reactions towards people who have mental illness.

| 1  (Strongly Disagree) | 2  (Disagree) | 3  (Neither Agree nor Disagree) | 4  (Agree) | 5  (Strongly Agree) |
| --- | --- | --- | --- | --- |

There is little I can do to help people with mental illness.

| 1  (Strongly Disagree) | 2  (Disagree) | 3  (Neither Agree nor Disagree) | 4  (Agree) | 5  (Strongly Agree) |
| --- | --- | --- | --- | --- |

More than half of people with mental illness don’t try hard enough to get better.

| 1  (Strongly Disagree) | 2  (Disagree) | 3  (Neither Agree nor Disagree) | 4  (Agree) | 5  (Strongly Agree) |
| --- | --- | --- | --- | --- |

I would not want a person with a mental illness, even if it were appropriately managed, to work with children.

| 1  (Strongly Disagree) | 2  (Disagree) | 3  (Neither Agree nor Disagree) | 4  (Agree) | 5  (Strongly Agree) |
| --- | --- | --- | --- | --- |

Healthcare providers do not need to be advocates for people with mental illness.

| 1  (Strongly Disagree) | 2  (Disagree) | 3  (Neither Agree nor Disagree) | 4  (Agree) | 5  (Strongly Agree) |
| --- | --- | --- | --- | --- |

I would not mind if a person with a mental illness lived next door to me.

| 1  (Strongly Disagree) | 2  (Disagree) | 3  (Neither Agree nor Disagree) | 4  (Agree) | 5  (Strongly Agree) |
| --- | --- | --- | --- | --- |

I struggle to feel compassion for a person with mental illness.

| 1  (Strongly Disagree) | 2  (Disagree) | 3  (Neither Agree nor Disagree) | 4  (Agree) | 5  (Strongly Agree) |
| --- | --- | --- | --- | --- |

**The following questions are for purposes of gaining an understanding of your knowledge of various aspects of mental health. When responding, we are interested in your degree of knowledge, When choosing your response, consider that:**

Very unlikely = I am certain that it is NOT likely

Unlikely = I think it is unlikely but am not certain

Likely = I think it is likely but am not certain

Very likely = I am certain that it IS very likely

If someone became extremely nervous or anxious in one or more situations with other people (e.g., a party) or performance situations (e.g., presenting at a meeting) in which they were afraid of being evaluated by others and that they would act in a way that was humiliating or feel embarrassed, then to what extent do you think it is likely they have **Social Phobia:**

| 1  (Very unlikely) | 2  (Unlikely) | 3  (Likely) | 4  (Very likely) |
| --- | --- | --- | --- |

If someone experienced excessive worry about a number of events or activities where this level of concern was not warranted, had difficulty controlling this worry and had physical symptoms such as having tense muscles and feeling fatigued then to what extent do you think it is likely they have Generalised Anxiety Disorder:

| 1  (Very unlikely) | 2  (Unlikely) | 3  (Likely) | 4  (Very likely) |
| --- | --- | --- | --- |

If someone experienced a low mood for two or more weeks, had a loss of pleasure or interest in their normal activities and experienced changes in their appetite and sleep then to what extent do you think it is likely they have **Major Depressive Disorder:**

| 1  (Very unlikely) | 2  (Unlikely) | 3  (Likely) | 4  (Very likely) |
| --- | --- | --- | --- |

To what extent do you think it is likely that **Personality Disorders** are a category of mental illness:

| 1  (Very unlikely) | 2  (Unlikely) | 3  (Likely) | 4  (Very likely) |
| --- | --- | --- | --- |

To what extent do you think it is likely that **Dysthymia** is a disorder:

| 1  (Very unlikely) | 2  (Unlikely) | 3  (Likely) | 4  (Very likely) |
| --- | --- | --- | --- |

To what extent do you think it is likely that the diagnosis of **Agoraphobia** includes anxiety about situations where escape may be difficult or embarrassing:

| 1  (Very unlikely) | 2  (Unlikely) | 3  (Likely) | 4  (Very likely) |
| --- | --- | --- | --- |

To what extent do you think it is likely that the diagnosis of **Bipolar Disorder** includes experiencing periods of elevated (i.e., high) and periods of depressed (i.e., low) mood:

| 1  (Very unlikely) | 2  (Unlikely) | 3  (Likely) | 4  (Very likely) |
| --- | --- | --- | --- |

To what extent do you think it is likely that the diagnosis of **Drug Dependence** includes physical and psychological tolerance of the drug (i.e., require more of the drug to get the same effect):

| 1  (Very unlikely) | 2  (Unlikely) | 3  (Likely) | 4  (Very likely) |
| --- | --- | --- | --- |

To what extent do you think it is likely that in general in Singapore, **women are MORE likely to experience a mental illness of any kind compared to men:**

| 1  (Very unlikely) | 2  (Unlikely) | 3  (Likely) | 4  (Very likely) |
| --- | --- | --- | --- |

To what extent do you think it is likely that in general, in Singapore **men are MORE likely to experience an anxiety disorder compared to women:**

| 1  (Very unlikely) | 2  (Unlikely) | 3  (Likely) | 4  (Very likely) |
| --- | --- | --- | --- |

**When choosing your response, consider that:**

**Very unhelpful = I am certain that it is NOT helpful**

**Unhelpful = I think it is unhelpful but am not certain**

**Helpful = I think it is helpful but am not certain**

**Very helpful = I am certain it IS very helpful**

To what extent do you think it would be helpful for someone to **improve their quality of sleep** if they were having difficulties managing their emotions (e.g., becoming very anxious or depressed):

| 1  (Very unhelpful) | 2  (Unhelpful) | 3  (Helpful) | 4  (Very helpful) |
| --- | --- | --- | --- |

To what extent do you think it would be helpful for someone to **avoid all activities or situations that made them feel anxious** if they were having difficulties managing their emotions:

| 1  (Very unhelpful) | 2  (Unhelpful) | 3  (Helpful) | 4  (Very helpful) |
| --- | --- | --- | --- |

When choosing your response, consider that:

Very unlikely = I am certain that it is NOT likely

Unlikely = I think it is unlikely but am not certain

Likely = I think it is likely but am not certain

Very likely = I am certain that it IS very likely

To what extent do you think it is likely that **Cognitive Behaviour Therapy (CBT)** is a therapy based on challenging negative thoughts and increasing helpful behaviours

| 1  (Very unlikely) | 2  (Unlikely) | 3  (Likely) | 4  (Very likely) |
| --- | --- | --- | --- |

**Mental health professionals are bound by confidentiality. However, there are certain conditions under which this does not apply.**

**To what extent do you think it is likely that the following condition that would allow a mental health professional to break confidentiality:**

***If you are at immediate risk of harm to yourself or others***

| 1  (Very unlikely) | 2  (Unlikely) | 3  (Likely) | 4  (Very likely) |
| --- | --- | --- | --- |

**Mental health professionals are bound by confidentiality. However, there are certain conditions under which this does not apply.**

**To what extent do you think it is likely that the following condition that would allow a mental health professional to break confidentiality:**

***If your problem is not life-threatening and they want to assist others to better support you***

| 1  (Very unlikely) | 2  (Unlikely) | 3  (Likely) | 4  (Very likely) |
| --- | --- | --- | --- |

**Please indicate to what extent you agree with the following statements:**

|  | Strongly Disagree | Disagree | Neither agree or disagree | Agree | Strongly agree |
| --- | --- | --- | --- | --- | --- |
| I am confident that I know where to seek information about mental illnesses. |  |  |  |  |  |
| I am confident using the computer or telephone to seek information about mental illnesses. |  |  |  |  |  |
| I am confident attending face to face appointments to seek information about mental illness (e.g., seeing the GP). |  |  |  |  |  |
| I am confident I have access to resources (e.g., GP, internet, friends) that I can use to seek information about mental illness. |  |  |  |  |  |
| People with a mental illness could snap out if it if they wanted. |  |  |  |  |  |
| A mental illness is a sign of personal weakness. |  |  |  |  |  |
| A mental illness is not a real medical illness. |  |  |  |  |  |
| People with a mental illness are dangerous. |  |  |  |  |  |
| It is best to avoid people with a mental illness so that you don't develop this problem. |  |  |  |  |  |
| If I had a mental illness I would not tell anyone. |  |  |  |  |  |
| Seeing a mental health professional means you are not strong enough to manage your own difficulties. |  |  |  |  |  |
| If I had a mental illness, I would not seek help from a mental health professional. |  |  |  |  |  |
| I believe treatment for a mental illness, provided by a mental health professional, would not be effective. |  |  |  |  |  |

Please indicate to what extent you agree with the following statements:

|  | Definitely unwilling | Probably unwilling | Neither unwilling or willing | Probably willing | Definitely willing |
| --- | --- | --- | --- | --- | --- |
| How willing would you be to move next door to someone with a mental illness? |  |  |  |  |  |
| How willing would you be to spend an evening socialising with someone with a mental illness? |  |  |  |  |  |
| How willing would you be to make friends with someone with a mental illness? |  |  |  |  |  |
| How willing would you be to have someone with a mental illness start working closely with you on a job? |  |  |  |  |  |
| How willing would you be to have someone with a mental illness marry into your family? |  |  |  |  |  |
| How willing would you be to vote for a politician if you knew they had suffered a mental illness? |  |  |  |  |  |
| How willing would you be to employ someone if you knew they had a mental illness? |  |  |  |  |  |

**The following questions are on your personal attitudes about empathy in the context of health professions education and patient care.**

Health care providers’ understanding of their patients’ feelings and the feelings of their patients’ families does not influence treatment outcomes.

| 1  (Strongly Disagree) | 2 | 3 | 4 | 5 | 6 | 7  (Strongly Agree) |
| --- | --- | --- | --- | --- | --- | --- |

Patients feel better when their healthcare providers understand their feelings.

| 1  (Strongly Disagree) | 2 | 3 | 4 | 5 | 6 | 7  (Strongly Agree) |
| --- | --- | --- | --- | --- | --- | --- |

It is difficult for a health care provider to view things from patients’ perspectives.

| 1  (Strongly Disagree) | 2 | 3 | 4 | 5 | 6 | 7  (Strongly Agree) |
| --- | --- | --- | --- | --- | --- | --- |

Understanding body language is as important as verbal communication in health care provider-patient relationships.

| 1  (Strongly Disagree) | 2 | 3 | 4 | 5 | 6 | 7  (Strongly Agree) |
| --- | --- | --- | --- | --- | --- | --- |

A health care provider’s sense of humour contributes to a better clinical outcome.

| 1  (Strongly Disagree) | 2 | 3 | 4 | 5 | 6 | 7  (Strongly Agree) |
| --- | --- | --- | --- | --- | --- | --- |

Because people are different, it is difficult to see things from patients’ perspectives.

| 1  (Strongly Disagree) | 2 | 3 | 4 | 5 | 6 | 7  (Strongly Agree) |
| --- | --- | --- | --- | --- | --- | --- |

Attention to patients’ emotions is not important in patient interview.

| 1  (Strongly Disagree) | 2 | 3 | 4 | 5 | 6 | 7  (Strongly Agree) |
| --- | --- | --- | --- | --- | --- | --- |

Attentiveness to patients’ personal experiences does not influence treatment outcomes.

| 1  (Strongly Disagree) | 2 | 3 | 4 | 5 | 6 | 7  (Strongly Agree) |
| --- | --- | --- | --- | --- | --- | --- |

Health care providers should try to stand in their patients’ shoes when providing care to them.

| 1  (Strongly Disagree) | 2 | 3 | 4 | 5 | 6 | 7  (Strongly Agree) |
| --- | --- | --- | --- | --- | --- | --- |

Patients value a health care provider’s understanding of their feelings which is therapeutic in its own right.

| 1  (Strongly Disagree) | 2 | 3 | 4 | 5 | 6 | 7  (Strongly Agree) |
| --- | --- | --- | --- | --- | --- | --- |

Patients’ illnesses can be cured only by targeted treatment; therefore, health care providers’ emotional ties with their patients do not have a significant influence in treatment outcomes.

| 1  (Strongly Disagree) | 2 | 3 | 4 | 5 | 6 | 7  (Strongly Agree) |
| --- | --- | --- | --- | --- | --- | --- |

Asking patients about what is happening in their personal lives is not helpful in understanding their physical complaints.

| 1  (Strongly Disagree) | 2 | 3 | 4 | 5 | 6 | 7  (Strongly Agree) |
| --- | --- | --- | --- | --- | --- | --- |

Health care providers should try to understand what is going on in their patients’ minds by paying attention to their non-verbal cues and body language.

| 1  (Strongly Disagree) | 2 | 3 | 4 | 5 | 6 | 7  (Strongly Agree) |
| --- | --- | --- | --- | --- | --- | --- |

I believe that emotion has no place in the treatment of medical illness.

| 1  (Strongly Disagree) | 2 | 3 | 4 | 5 | 6 | 7  (Strongly Agree) |
| --- | --- | --- | --- | --- | --- | --- |

Empathy is a therapeutic skill without which the health care provider’s success is limited.

| 1  (Strongly Disagree) | 2 | 3 | 4 | 5 | 6 | 7  (Strongly Agree) |
| --- | --- | --- | --- | --- | --- | --- |

Health care providers’ understanding of the emotional status of their parents, as well as that of their families is one important component of the health care provider-patient relationship.

| 1  (Strongly Disagree) | 2 | 3 | 4 | 5 | 6 | 7  (Strongly Agree) |
| --- | --- | --- | --- | --- | --- | --- |

Health care providers should try to think like their patients in order to render better care.

| 1  (Strongly Disagree) | 2 | 3 | 4 | 5 | 6 | 7  (Strongly Agree) |
| --- | --- | --- | --- | --- | --- | --- |

Health care providers should not allow themselves to be influenced by strong personal bonds between their patients and their family members.

| 1  (Strongly Disagree) | 2 | 3 | 4 | 5 | 6 | 7  (Strongly Agree) |
| --- | --- | --- | --- | --- | --- | --- |

I do not enjoy reading non-medical literature or the arts.

| 1  (Strongly Disagree) | 2 | 3 | 4 | 5 | 6 | 7  (Strongly Agree) |
| --- | --- | --- | --- | --- | --- | --- |

I believe that empathy is an important therapeutic factor in patients’ treatment.

| 1  (Strongly Disagree) | 2 | 3 | 4 | 5 | 6 | 7  (Strongly Agree) |
| --- | --- | --- | --- | --- | --- | --- |

**Annex D. Post-MAGIC Questionnaire for Nursing Students**

Please provide the last four digits of your mobile number for us to do matching of your responses.

|  |  |  |  |
| --- | --- | --- | --- |

**The following questions are on your personal experiences with agitated patients.**

If given a choice, would you treat a patient with mental illness?

Yes

No

How confident are you in managing patients who are agitated?

| 1  (Least) | 2 | 3 | 4 | 5 | 6 | 7  (Most) |
| --- | --- | --- | --- | --- | --- | --- |

How confident are you in using verbal de-escalation techniques to manage patients who are agitated?

| 1  (Least) | 2 | 3 | 4 | 5 | 6 | 7  (Most) |
| --- | --- | --- | --- | --- | --- | --- |

How confident are you in using chemical tranquilisation to manage patients who are agitated?

| 1  (Least) | 2 | 3 | 4 | 5 | 6 | 7  (Most) |
| --- | --- | --- | --- | --- | --- | --- |

How confident are you in using physical restraint to manage patients who are agitated?

| 1  (Least) | 2 | 3 | 4 | 5 | 6 | 7  (Most) |
| --- | --- | --- | --- | --- | --- | --- |

How confident are you in communicating with an agitated person?

| 1  (Least) | 2 | 3 | 4 | 5 | 6 | 7  (Most) |
| --- | --- | --- | --- | --- | --- | --- |

How confident are you in managing a situation (including the environment, other people etc) that involves an agitated patient?

| 1  (Least) | 2 | 3 | 4 | 5 | 6 | 7  (Most) |
| --- | --- | --- | --- | --- | --- | --- |

How would you rate your level of empathy towards patients?

| 1  (Least) | 2 | 3 | 4 | 5 | 6 | 7  (Most) |
| --- | --- | --- | --- | --- | --- | --- |

I am ready to provide care for an agitated patient.

| 1  (Least) | 2 | 3 | 4 | 5 | 6 | 7  (Most) |
| --- | --- | --- | --- | --- | --- | --- |

I am competent in caring for patients with mental health conditions.

| 1  (Least) | 2 | 3 | 4 | 5 | 6 | 7  (Most) |
| --- | --- | --- | --- | --- | --- | --- |

I am competent in managing agitated patients.

| 1  (Least) | 2 | 3 | 4 | 5 | 6 | 7  (Most) |
| --- | --- | --- | --- | --- | --- | --- |

**The following questions assess attitudes and behavioural intentions towards people with mental illness. There is no correct answer. Please mark the box that best fits your opinion.**

I am more comfortable helping a person who has a physical illness than I am helping a person who has a mental illness.

| 1  (Strongly Disagree) | 2  (Disagree) | 3  (Neither Agree nor Disagree) | 4  (Agree) | 5  (Strongly Agree) |
| --- | --- | --- | --- | --- |

If a colleague with who I work with told me they had a mental illness, I would be just as willing to work with him / her.

| 1  (Strongly Disagree) | 2  (Disagree) | 3  (Neither Agree nor Disagree) | 4  (Agree) | 5  (Strongly Agree) |
| --- | --- | --- | --- | --- |

If I were under treatment for a mental illness, I would not disclose this to any of my colleagues.

| 1  (Strongly Disagree) | 2  (Disagree) | 3  (Neither Agree nor Disagree) | 4  (Agree) | 5  (Strongly Agree) |
| --- | --- | --- | --- | --- |

I would see myself as weak if I had a mental illness and could not fix it myself.

| 1  (Strongly Disagree) | 2  (Disagree) | 3  (Neither Agree nor Disagree) | 4  (Agree) | 5  (Strongly Agree) |
| --- | --- | --- | --- | --- |

I would be reluctant to seek help if I had a mental illness.

| 1  (Strongly Disagree) | 2  (Disagree) | 3  (Neither Agree nor Disagree) | 4  (Agree) | 5  (Strongly Agree) |
| --- | --- | --- | --- | --- |

Employers should hire a person with a managed mental illness if he / she is the best person for the job.

| 1  (Strongly Disagree) | 2  (Disagree) | 3  (Neither Agree nor Disagree) | 4  (Agree) | 5  (Strongly Agree) |
| --- | --- | --- | --- | --- |

I would still go to a physician if I knew that the physician had been treated for a mental illness.

| 1  (Strongly Disagree) | 2  (Disagree) | 3  (Neither Agree nor Disagree) | 4  (Agree) | 5  (Strongly Agree) |
| --- | --- | --- | --- | --- |

If I had a mental illness, I would tell my friends.

| 1  (Strongly Disagree) | 2  (Disagree) | 3  (Neither Agree nor Disagree) | 4  (Agree) | 5  (Strongly Agree) |
| --- | --- | --- | --- | --- |

Despite my professional beliefs, I have negative reactions towards people who have mental illness.

| 1  (Strongly Disagree) | 2  (Disagree) | 3  (Neither Agree nor Disagree) | 4  (Agree) | 5  (Strongly Agree) |
| --- | --- | --- | --- | --- |

There is little I can do to help people with mental illness.

| 1  (Strongly Disagree) | 2  (Disagree) | 3  (Neither Agree nor Disagree) | 4  (Agree) | 5  (Strongly Agree) |
| --- | --- | --- | --- | --- |

More than half of people with mental illness don’t try hard enough to get better.

| 1  (Strongly Disagree) | 2  (Disagree) | 3  (Neither Agree nor Disagree) | 4  (Agree) | 5  (Strongly Agree) |
| --- | --- | --- | --- | --- |

I would not want a person with a mental illness, even if it were appropriately managed, to work with children.

| 1  (Strongly Disagree) | 2  (Disagree) | 3  (Neither Agree nor Disagree) | 4  (Agree) | 5  (Strongly Agree) |
| --- | --- | --- | --- | --- |

Healthcare providers do not need to be advocates for people with mental illness.

| 1  (Strongly Disagree) | 2  (Disagree) | 3  (Neither Agree nor Disagree) | 4  (Agree) | 5  (Strongly Agree) |
| --- | --- | --- | --- | --- |

I would not mind if a person with a mental illness lived next door to me.

| 1  (Strongly Disagree) | 2  (Disagree) | 3  (Neither Agree nor Disagree) | 4  (Agree) | 5  (Strongly Agree) |
| --- | --- | --- | --- | --- |

I struggle to feel compassion for a person with mental illness.

| 1  (Strongly Disagree) | 2  (Disagree) | 3  (Neither Agree nor Disagree) | 4  (Agree) | 5  (Strongly Agree) |
| --- | --- | --- | --- | --- |

**The following questions are for purposes of gaining an understanding of your knowledge of various aspects of mental health. When responding, we are interested in your degree of knowledge, When choosing your response, consider that:**

Very unlikely = I am certain that it is NOT likely

Unlikely = I think it is unlikely but am not certain

Likely = I think it is likely but am not certain

Very likely = I am certain that it IS very likely

If someone became extremely nervous or anxious in one or more situations with other people (e.g., a party) or performance situations (e.g., presenting at a meeting) in which they were afraid of being evaluated by others and that they would act in a way that was humiliating or feel embarrassed, then to what extent do you think it is likely they have **Social Phobia:**

| 1  (Very unlikely) | 2  (Unlikely) | 3  (Likely) | 4  (Very likely) |
| --- | --- | --- | --- |

If someone experienced excessive worry about a number of events or activities where this level of concern was not warranted, had difficulty controlling this worry and had physical symptoms such as having tense muscles and feeling fatigued then to what extent do you think it is likely they have Generalised Anxiety Disorder:

| 1  (Very unlikely) | 2  (Unlikely) | 3  (Likely) | 4  (Very likely) |
| --- | --- | --- | --- |

If someone experienced a low mood for two or more weeks, had a loss of pleasure or interest in their normal activities and experienced changes in their appetite and sleep then to what extent do you think it is likely they have **Major Depressive Disorder:**

| 1  (Very unlikely) | 2  (Unlikely) | 3  (Likely) | 4  (Very likely) |
| --- | --- | --- | --- |

To what extent do you think it is likely that **Personality Disorders** are a category of mental illness:

| 1  (Very unlikely) | 2  (Unlikely) | 3  (Likely) | 4  (Very likely) |
| --- | --- | --- | --- |

To what extent do you think it is likely that **Dysthymia** is a disorder:

| 1  (Very unlikely) | 2  (Unlikely) | 3  (Likely) | 4  (Very likely) |
| --- | --- | --- | --- |

To what extent do you think it is likely that the diagnosis of **Agoraphobia** includes anxiety about situations where escape may be difficult or embarrassing:

| 1  (Very unlikely) | 2  (Unlikely) | 3  (Likely) | 4  (Very likely) |
| --- | --- | --- | --- |

To what extent do you think it is likely that the diagnosis of **Bipolar Disorder** includes experiencing periods of elevated (i.e., high) and periods of depressed (i.e., low) mood:

| 1  (Very unlikely) | 2  (Unlikely) | 3  (Likely) | 4  (Very likely) |
| --- | --- | --- | --- |

To what extent do you think it is likely that the diagnosis of **Drug Dependence** includes physical and psychological tolerance of the drug (i.e., require more of the drug to get the same effect):

| 1  (Very unlikely) | 2  (Unlikely) | 3  (Likely) | 4  (Very likely) |
| --- | --- | --- | --- |

To what extent do you think it is likely that in general in Singapore, **women are MORE likely to experience a mental illness of any kind compared to men:**

| 1  (Very unlikely) | 2  (Unlikely) | 3  (Likely) | 4  (Very likely) |
| --- | --- | --- | --- |

To what extent do you think it is likely that in general, in Singapore **men are MORE likely to experience an anxiety disorder compared to women:**

| 1  (Very unlikely) | 2  (Unlikely) | 3  (Likely) | 4  (Very likely) |
| --- | --- | --- | --- |

**When choosing your response, consider that:**

**Very unhelpful = I am certain that it is NOT helpful**

**Unhelpful = I think it is unhelpful but am not certain**

**Helpful = I think it is helpful but am not certain**

**Very helpful = I am certain it IS very helpful**

To what extent do you think it would be helpful for someone to **improve their quality of sleep** if they were having difficulties managing their emotions (e.g., becoming very anxious or depressed):

| 1  (Very unhelpful) | 2  (Unhelpful) | 3  (Helpful) | 4  (Very helpful) |
| --- | --- | --- | --- |

To what extent do you think it would be helpful for someone to **avoid all activities or situations that made them feel anxious** if they were having difficulties managing their emotions:

| 1  (Very unhelpful) | 2  (Unhelpful) | 3  (Helpful) | 4  (Very helpful) |
| --- | --- | --- | --- |

When choosing your response, consider that:

Very unlikely = I am certain that it is NOT likely

Unlikely = I think it is unlikely but am not certain

Likely = I think it is likely but am not certain

Very likely = I am certain that it IS very likely

To what extent do you think it is likely that **Cognitive Behaviour Therapy (CBT)** is a therapy based on challenging negative thoughts and increasing helpful behaviours

| 1  (Very unlikely) | 2  (Unlikely) | 3  (Likely) | 4  (Very likely) |
| --- | --- | --- | --- |

**Mental health professionals are bound by confidentiality. However, there are certain conditions under which this does not apply.**

**To what extent do you think it is likely that the following condition that would allow a mental health professional to break confidentiality:**

***If you are at immediate risk of harm to yourself or others***

| 1  (Very unlikely) | 2  (Unlikely) | 3  (Likely) | 4  (Very likely) |
| --- | --- | --- | --- |

**Mental health professionals are bound by confidentiality. However, there are certain conditions under which this does not apply.**

**To what extent do you think it is likely that the following condition that would allow a mental health professional to break confidentiality:**

***If your problem is not life-threatening and they want to assist others to better support you***

| 1  (Very unlikely) | 2  (Unlikely) | 3  (Likely) | 4  (Very likely) |
| --- | --- | --- | --- |

**Please indicate to what extent you agree with the following statements:**

|  | Strongly Disagree | Disagree | Neither agree or disagree | Agree | Strongly agree |
| --- | --- | --- | --- | --- | --- |
| I am confident that I know where to seek information about mental illnesses. |  |  |  |  |  |
| I am confident using the computer or telephone to seek information about mental illnesses. |  |  |  |  |  |
| I am confident attending face to face appointments to seek information about mental illness (e.g., seeing the GP). |  |  |  |  |  |
| I am confident I have access to resources (e.g., GP, internet, friends) that I can use to seek information about mental illness. |  |  |  |  |  |
| People with a mental illness could snap out if it if they wanted. |  |  |  |  |  |
| A mental illness is a sign of personal weakness. |  |  |  |  |  |
| A mental illness is not a real medical illness. |  |  |  |  |  |
| People with a mental illness are dangerous. |  |  |  |  |  |
| It is best to avoid people with a mental illness so that you don't develop this problem. |  |  |  |  |  |
| If I had a mental illness I would not tell anyone. |  |  |  |  |  |
| Seeing a mental health professional means you are not strong enough to manage your own difficulties. |  |  |  |  |  |
| If I had a mental illness, I would not seek help from a mental health professional. |  |  |  |  |  |
| I believe treatment for a mental illness, provided by a mental health professional, would not be effective. |  |  |  |  |  |

Please indicate to what extent you agree with the following statements:

|  | Definitely unwilling | Probably unwilling | Neither unwilling or willing | Probably willing | Definitely willing |
| --- | --- | --- | --- | --- | --- |
| How willing would you be to move next door to someone with a mental illness? |  |  |  |  |  |
| How willing would you be to spend an evening socialising with someone with a mental illness? |  |  |  |  |  |
| How willing would you be to make friends with someone with a mental illness? |  |  |  |  |  |
| How willing would you be to have someone with a mental illness start working closely with you on a job? |  |  |  |  |  |
| How willing would you be to have someone with a mental illness marry into your family? |  |  |  |  |  |
| How willing would you be to vote for a politician if you knew they had suffered a mental illness? |  |  |  |  |  |
| How willing would you be to employ someone if you knew they had a mental illness? |  |  |  |  |  |

**The following questions are on your personal attitudes about empathy in the context of health professions education and patient care.**

Health care providers’ understanding of their patients’ feelings and the feelings of their patients’ families does not influence treatment outcomes.

| 1  (Strongly Disagree) | 2 | 3 | 4 | 5 | 6 | 7  (Strongly Agree) |
| --- | --- | --- | --- | --- | --- | --- |

Patients feel better when their healthcare providers understand their feelings.

| 1  (Strongly Disagree) | 2 | 3 | 4 | 5 | 6 | 7  (Strongly Agree) |
| --- | --- | --- | --- | --- | --- | --- |

It is difficult for a health care provider to view things from patients’ perspectives.

| 1  (Strongly Disagree) | 2 | 3 | 4 | 5 | 6 | 7  (Strongly Agree) |
| --- | --- | --- | --- | --- | --- | --- |

Understanding body language is as important as verbal communication in health care provider-patient relationships.

| 1  (Strongly Disagree) | 2 | 3 | 4 | 5 | 6 | 7  (Strongly Agree) |
| --- | --- | --- | --- | --- | --- | --- |

A health care provider’s sense of humour contributes to a better clinical outcome.

| 1  (Strongly Disagree) | 2 | 3 | 4 | 5 | 6 | 7  (Strongly Agree) |
| --- | --- | --- | --- | --- | --- | --- |

Because people are different, it is difficult to see things from patients’ perspectives.

| 1  (Strongly Disagree) | 2 | 3 | 4 | 5 | 6 | 7  (Strongly Agree) |
| --- | --- | --- | --- | --- | --- | --- |

Attention to patients’ emotions is not important in patient interview.

| 1  (Strongly Disagree) | 2 | 3 | 4 | 5 | 6 | 7  (Strongly Agree) |
| --- | --- | --- | --- | --- | --- | --- |

Attentiveness to patients’ personal experiences does not influence treatment outcomes.

| 1  (Strongly Disagree) | 2 | 3 | 4 | 5 | 6 | 7  (Strongly Agree) |
| --- | --- | --- | --- | --- | --- | --- |

Health care providers should try to stand in their patients’ shoes when providing care to them.

| 1  (Strongly Disagree) | 2 | 3 | 4 | 5 | 6 | 7  (Strongly Agree) |
| --- | --- | --- | --- | --- | --- | --- |

Patients value a health care provider’s understanding of their feelings which is therapeutic in its own right.

| 1  (Strongly Disagree) | 2 | 3 | 4 | 5 | 6 | 7  (Strongly Agree) |
| --- | --- | --- | --- | --- | --- | --- |

Patients’ illnesses can be cured only by targeted treatment; therefore, health care providers’ emotional ties with their patients do not have a significant influence in treatment outcomes.

| 1  (Strongly Disagree) | 2 | 3 | 4 | 5 | 6 | 7  (Strongly Agree) |
| --- | --- | --- | --- | --- | --- | --- |

Asking patients about what is happening in their personal lives is not helpful in understanding their physical complaints.

| 1  (Strongly Disagree) | 2 | 3 | 4 | 5 | 6 | 7  (Strongly Agree) |
| --- | --- | --- | --- | --- | --- | --- |

Health care providers should try to understand what is going on in their patients’ minds by paying attention to their non-verbal cues and body language.

| 1  (Strongly Disagree) | 2 | 3 | 4 | 5 | 6 | 7  (Strongly Agree) |
| --- | --- | --- | --- | --- | --- | --- |

I believe that emotion has no place in the treatment of medical illness.

| 1  (Strongly Disagree) | 2 | 3 | 4 | 5 | 6 | 7  (Strongly Agree) |
| --- | --- | --- | --- | --- | --- | --- |

Empathy is a therapeutic skill without which the health care provider’s success is limited.

| 1  (Strongly Disagree) | 2 | 3 | 4 | 5 | 6 | 7  (Strongly Agree) |
| --- | --- | --- | --- | --- | --- | --- |

Health care providers’ understanding of the emotional status of their parents, as well as that of their families is one important component of the health care provider-patient relationship.

| 1  (Strongly Disagree) | 2 | 3 | 4 | 5 | 6 | 7  (Strongly Agree) |
| --- | --- | --- | --- | --- | --- | --- |

Health care providers should try to think like their patients in order to render better care.

| 1  (Strongly Disagree) | 2 | 3 | 4 | 5 | 6 | 7  (Strongly Agree) |
| --- | --- | --- | --- | --- | --- | --- |

Health care providers should not allow themselves to be influenced by strong personal bonds between their patients and their family members.

| 1  (Strongly Disagree) | 2 | 3 | 4 | 5 | 6 | 7  (Strongly Agree) |
| --- | --- | --- | --- | --- | --- | --- |

I do not enjoy reading non-medical literature or the arts.

| 1  (Strongly Disagree) | 2 | 3 | 4 | 5 | 6 | 7  (Strongly Agree) |
| --- | --- | --- | --- | --- | --- | --- |

I believe that empathy is an important therapeutic factor in patients’ treatment.

| 1  (Strongly Disagree) | 2 | 3 | 4 | 5 | 6 | 7  (Strongly Agree) |
| --- | --- | --- | --- | --- | --- | --- |

**The following questions are on your experience with using the VR software.**

What is the level of immersion you experienced?

| 1  (Extremely Low) | 2  (Very Low) | 3  (Low) | 4  (Neutral) | 5  (High) | 6  (Very High) | 7  (Extremely High) |
| --- | --- | --- | --- | --- | --- | --- |

Any additional comments and / or suggestions relevant to the question above:

|  |
| --- |

What was your level of enjoyment of the VR experience?

| 1  (Extremely Low) | 2  (Very Low) | 3  (Low) | 4  (Neutral) | 5  (High) | 6  (Very High) | 7  (Extremely High) |
| --- | --- | --- | --- | --- | --- | --- |

Any additional comments and / or suggestions relevant to the question above:

|  |
| --- |

How was the quality of the graphics?

| 1  (Extremely Low) | 2  (Very Low) | 3  (Low) | 4  (Neutral) | 5  (High) | 6  (Very High) | 7  (Extremely High) |
| --- | --- | --- | --- | --- | --- | --- |

Any additional comments and / or suggestions relevant to the question above:

|  |
| --- |

How was the quality of sound?

| 1  (Extremely Low) | 2  (Very Low) | 3  (Low) | 4  (Neutral) | 5  (High) | 6  (Very High) | 7  (Extremely High) |
| --- | --- | --- | --- | --- | --- | --- |

Any additional comments and / or suggestions relevant to the question above:

|  |
| --- |

How was the quality of the VR technology overall (i.e., hardware and peripherals)?

| 1  (Extremely Low) | 2  (Very Low) | 3  (Low) | 4  (Neutral) | 5  (High) | 6  (Very High) | 7  (Extremely High) |
| --- | --- | --- | --- | --- | --- | --- |

Any additional comments and / or suggestions relevant to the question above:

|  |
| --- |

How easy was it to use the navigation system (e.g., teleportation) in the virtual environment?

| 1  (Extremely Low) | 2  (Very Low) | 3  (Low) | 4  (Neutral) | 5  (High) | 6  (Very High) | 7  (Extremely High) |
| --- | --- | --- | --- | --- | --- | --- |

Any additional comments and / or suggestions relevant to the question above:

|  |
| --- |

How easy was it to physically move in the virtual environment?

| 1  (Extremely Low) | 2  (Very Low) | 3  (Low) | 4  (Neutral) | 5  (High) | 6  (Very High) | 7  (Extremely High) |
| --- | --- | --- | --- | --- | --- | --- |

Any additional comments and / or suggestions relevant to the question above:

|  |
| --- |

How easy was it to pick up and / or place items in the virtual environment?

| 1  (Extremely Low) | 2  (Very Low) | 3  (Low) | 4  (Neutral) | 5  (High) | 6  (Very High) | 7  (Extremely High) |
| --- | --- | --- | --- | --- | --- | --- |

Any additional comments and / or suggestions relevant to the question above:

|  |
| --- |

How easy was it to use items in the virtual environment?

| 1  (Extremely Low) | 2  (Very Low) | 3  (Low) | 4  (Neutral) | 5  (High) | 6  (Very High) | 7  (Extremely High) |
| --- | --- | --- | --- | --- | --- | --- |

Any additional comments and / or suggestions relevant to the question above:

|  |
| --- |

How easy was the two-handed interaction e.g., grab the tablet with one hand, and push the button with the other hand?

| 1  (Extremely Low) | 2  (Very Low) | 3  (Low) | 4  (Neutral) | 5  (High) | 6  (Very High) | 7  (Extremely High) |
| --- | --- | --- | --- | --- | --- | --- |

Any additional comments and / or suggestions relevant to the question above:

|  |
| --- |

How easy was it to complete the tutorial?

| 1  (Extremely Low) | 2  (Very Low) | 3  (Low) | 4  (Neutral) | 5  (High) | 6  (Very High) | 7  (Extremely High) |
| --- | --- | --- | --- | --- | --- | --- |

Any additional comments and / or suggestions relevant to the question above:

|  |
| --- |

How helpful was / were the tutorial(s)?

| 1  (Extremely Low) | 2  (Very Low) | 3  (Low) | 4  (Neutral) | 5  (High) | 6  (Very High) | 7  (Extremely High) |
| --- | --- | --- | --- | --- | --- | --- |

Any additional comments and / or suggestions relevant to the question above:

|  |
| --- |

How did you feel about the duration of the tutorial?

| 1  (Extremely Low) | 2  (Very Low) | 3  (Low) | 4  (Neutral) | 5  (High) | 6  (Very High) | 7  (Extremely High) |
| --- | --- | --- | --- | --- | --- | --- |

Any additional comments and / or suggestions relevant to the question above:

|  |
| --- |

How helpful were the in-game instructions for the task you needed to perform?

| 1  (Extremely Low) | 2  (Very Low) | 3  (Low) | 4  (Neutral) | 5  (High) | 6  (Very High) | 7  (Extremely High) |
| --- | --- | --- | --- | --- | --- | --- |

Any additional comments and / or suggestions relevant to the question above:

|  |
| --- |

How helpful were the in-game prompts e.g., arrows showing the direction or labels?

| 1  (Extremely Low) | 2  (Very Low) | 3  (Low) | 4  (Neutral) | 5  (High) | 6  (Very High) | 7  (Extremely High) |
| --- | --- | --- | --- | --- | --- | --- |

Any additional comments and / or suggestions relevant to the question above:

|  |
| --- |

Did you experience nausea?

| 1  (Extremely Low) | 2  (Very Low) | 3  (Low) | 4  (Neutral) | 5  (High) | 6  (Very High) | 7  (Extremely High) |
| --- | --- | --- | --- | --- | --- | --- |

Any additional comments and / or suggestions relevant to the question above:

|  |
| --- |

Did you experience disorientation?

| 1  (Extremely Low) | 2  (Very Low) | 3  (Low) | 4  (Neutral) | 5  (High) | 6  (Very High) | 7  (Extremely High) |
| --- | --- | --- | --- | --- | --- | --- |

Any additional comments and / or suggestions relevant to the question above:

|  |
| --- |

Did you experience dizziness?

| 1  (Extremely Low) | 2  (Very Low) | 3  (Low) | 4  (Neutral) | 5  (High) | 6  (Very High) | 7  (Extremely High) |
| --- | --- | --- | --- | --- | --- | --- |

Any additional comments and / or suggestions relevant to the question above:

|  |
| --- |

Did you experience fatigue?

| 1  (Extremely Low) | 2  (Very Low) | 3  (Low) | 4  (Neutral) | 5  (High) | 6  (Very High) | 7  (Extremely High) |
| --- | --- | --- | --- | --- | --- | --- |

Any additional comments and / or suggestions relevant to the question above:

|  |
| --- |

Did you experience instability?

| 1  (Extremely Low) | 2  (Very Low) | 3  (Low) | 4  (Neutral) | 5  (High) | 6  (Very High) | 7  (Extremely High) |
| --- | --- | --- | --- | --- | --- | --- |

Any additional comments and / or suggestions relevant to the question above:

|  |
| --- |

**The following questions are on your experiences during the session.**

The VR software helped me understand symptoms faced by a patient with mental health conditions in a realistic manner.

| 1  (Strongly Disagree) | 2 | 3 | 4 | 5 | 6 | 7  (Strongly Agree) |
| --- | --- | --- | --- | --- | --- | --- |

The scenarios depicted are realistic and have learning values.

| 1  (Strongly Disagree) | 2 | 3 | 4 | 5 | 6 | 7  (Strongly Agree) |
| --- | --- | --- | --- | --- | --- | --- |

The VR software provided an immersive experience.

| 1  (Strongly Disagree) | 2 | 3 | 4 | 5 | 6 | 7  (Strongly Agree) |
| --- | --- | --- | --- | --- | --- | --- |

I would like to recommend other people to go through this experience because I find it helpful.

| 1  (Strongly Disagree) | 2 | 3 | 4 | 5 | 6 | 7  (Strongly Agree) |
| --- | --- | --- | --- | --- | --- | --- |

The session was conducted in an engaging manner.

| 1  (Strongly Disagree) | 2 | 3 | 4 | 5 | 6 | 7  (Strongly Agree) |
| --- | --- | --- | --- | --- | --- | --- |

The debrief session was useful for consolidating my learning.

| 1  (Strongly Disagree) | 2 | 3 | 4 | 5 | 6 | 7  (Strongly Agree) |
| --- | --- | --- | --- | --- | --- | --- |

Why was the debrief session useful / not useful?

|  |
| --- |

I would like to use the VR software again.

| 1  (Strongly Disagree) | 2 | 3 | 4 | 5 | 6 | 7  (Strongly Agree) |
| --- | --- | --- | --- | --- | --- | --- |

I now have a better understanding of how to manage agitated patients.

| 1  (Strongly Disagree) | 2 | 3 | 4 | 5 | 6 | 7  (Strongly Agree) |
| --- | --- | --- | --- | --- | --- | --- |

The VR software allowed me to learn how to manage agitated patients and psychiatric patients more efficiently than just attending didactic lectures and tutorials.

| 1  (Strongly Disagree) | 2 | 3 | 4 | 5 | 6 | 7  (Strongly Agree) |
| --- | --- | --- | --- | --- | --- | --- |

Please highlight the strengths / weaknesses of this session.

|  |
| --- |

Suggestions on how to improve this session.

|  |
| --- |

What other scenarios would you like to experience through the use of VR software?

|  |
| --- |

Any other comments?

|  |
| --- |


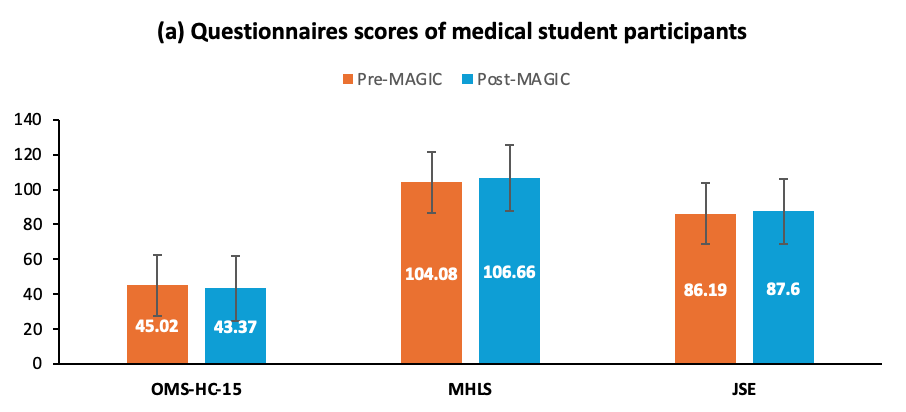

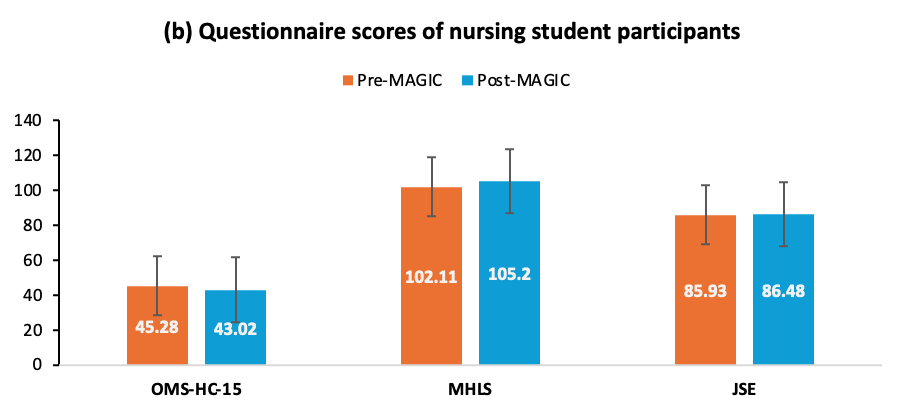


**Supplementary Figure 1**. Comparison between the questionnaire scores of (a) fourth-year medical students (N = 105), and (b) second-year nursing students (N = 47) pre- and post-MAGIC respectively.

*

*

*

*


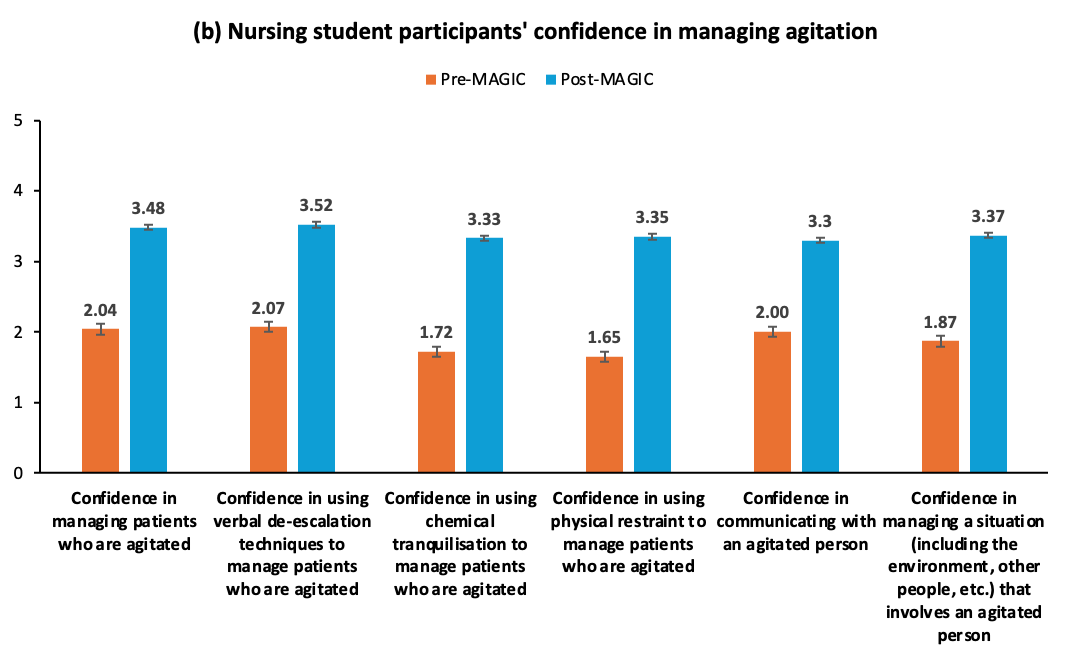

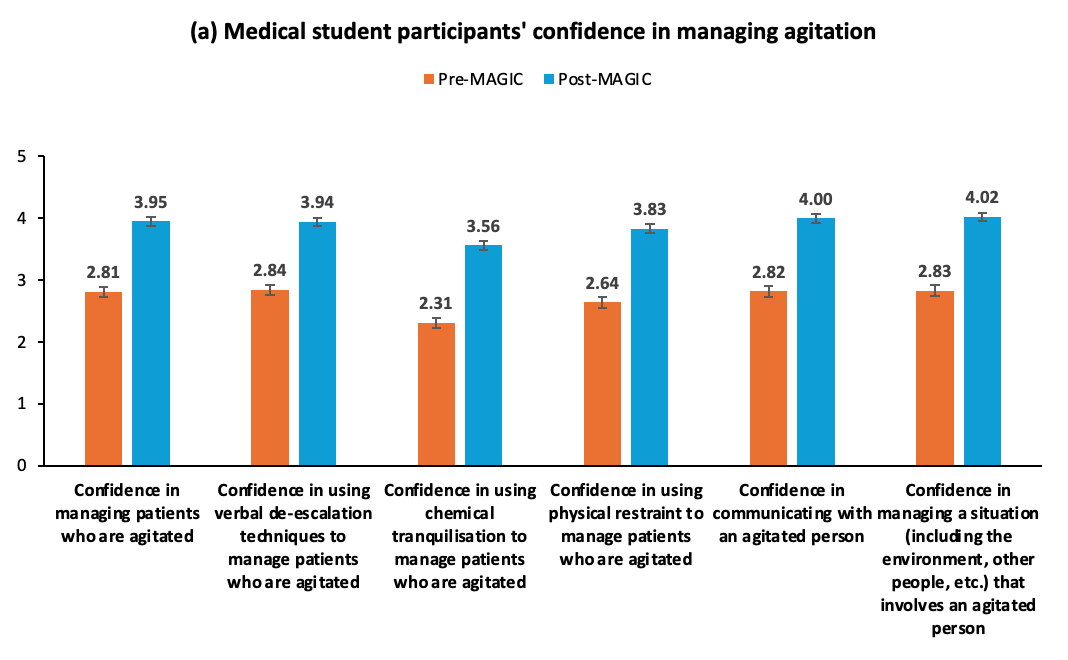


**Supplementary Figure 2.** Comparison between confidence levels of (a) fourth-year medical students (N = 105), and (b) second-year nursing students (N = 47) pre- and post-MAGIC.

*

*

*

*

*

*

*

*

*

*

*

*
